# An atlas of rabbit development as a model for single-cell comparative genomics

**DOI:** 10.1038/s41556-023-01174-0

**Published:** 2023-06-15

**Authors:** Mai-Linh Nu Ton, Daniel Keitley, Bart Theeuwes, Carolina Guibentif, Jonas Ahnfelt-Rønne, Thomas Kjærgaard Andreassen, Fernando J. Calero-Nieto, Ivan Imaz-Rosshandler, Blanca Pijuan-Sala, Jennifer Nichols, Èlia Benito-Gutiérrez, John C. Marioni, Berthold Göttgens

**Affiliations:** 1 Department of Haematology, University of Cambridge, Cambridge, UK; 2 Wellcome-Medical Research Council Cambridge Stem Cell Institute, University of Cambridge, Cambridge, UK; 3 Department of Zoology, University of Cambridge, Cambridge, UK; 4 Inst. Biomedicine, Dept. Microbiology and Immunology, Sahlgrenska Center for Cancer Research, University of Gothenburg, Gothenburg, Sweden; 5 Global Discovery & Development Sciences, Novo Nordisk, Måløv, Denmark; 6 Medical Research Council Laboratory of Molecular Biology, Cambridge, UK; 7 Genome Biology Unit, European Molecular Biology Laboratory (EMBL), Heidelberg, Germany; 8 MRC Human Genetics Unit, Institute of Genetics and Cancer, University of Edinburgh, Edinburgh, UK; 9 Wellcome Sanger Institute, Wellcome Genome Campus, Cambridge, UK; 10 European Molecular Biology Laboratory, European Bioinformatics Institute, Cambridge, UK; 11 Cancer Research UK Cambridge Institute, University of Cambridge Cambridge, UK

## Abstract

Traditionally, the mouse has been the favored vertebrate model for biomedical research, due to its experimental and genetic tractability. However, non-rodent embryological studies highlight that many aspects of early mouse development, such as its egg-cylinder gastrulation and method of implantation, diverge from other mammals, thus complicating inferences about human development. Like the human embryo, rabbits develop as a flat-bilaminar disc. In this study, we constructed a morphological and molecular atlas of rabbit development. We report transcriptional and chromatin accessibility profiles for over 180,000 single cells and high-resolution histology sections from embryos spanning gastrulation, implantation, amniogenesis, and early organogenesis. Using a neighborhood comparison pipeline, we compare the transcriptional landscape of rabbit and mouse at the scale of the entire organism. We characterize the gene regulatory programs underlying trophoblast differentiation and identify signaling interactions involving the yolk sac mesothelium during hematopoiesis. We demonstrate how the combination of both rabbit and mouse atlases can be leveraged to extract new biological insights from sparse macaque and human data. The datasets and computational pipelines reported here set a framework for a broader cross-species approach to decipher early mammalian development, and are readily adaptable to deploy single cell comparative genomics more broadly across biomedical research.

## Introduction

Due to the ethical and technical challenges of experimenting with human and non-human primate embryos, our understanding of human development is largely influenced by studies on the mouse. The mouse has dominated as a mammalian model for over 50 years; for its ease of maintenance and genetic manipulation, short generation times and large litter sizes. However, despite the utility of the mouse model in recapitulating many aspects of human biology, it has long been recognized that there is significant variability in early development between mammals and particularly between rodent and non-rodent species. A prominent example is the egg-cylinder shape of the mouse embryo, which substantially deviates from the flat-disc morphology of most other amniotes, including humans, and even other rodents^1^. The mouse embryo also diverges in other respects, such as the topology of its extra-embryonic tissues^2^ and method of implantation^3^.

Despite this, and although rabbits, dogs, sheep, pigs, and non-human primates are all used in biomedical research, the availability of deep molecular data in mammals is largely limited to the mouse. Previous molecular studies have characterized, at single-cell resolution, gastrulation and subsequent organogenesis in the mouse^4–7^. However, with advances in genome engineering and molecular profiling technologies^8^, more organisms have become accessible to functional experimentation and disease modeling. This presents an opportunity to gain a comparative understanding of species at the molecular and cellular level, which will be critical to determine optimal model systems, improve the translation of animal studies, and gain deeper insights into early mammalian development more broadly.

As an alternative to the mouse, the European rabbit, *Oryctolagus cuniculus*, offers many advantages for studying early mammalian development. Like mice, rabbits have short reproductive cycles (31 days), large litter sizes, and are well-established as a laboratory animal in pharmacological, reproductive, and developmental research^2,9–13^. Relative to mice, the rabbit embryo is highly accessible at later stages of development due to its large size and late implantation^12^. The embryo implants superficially, rather than intrusively embedding into the uterine lining, making it tractable to obtain embryos and extra-embryonic tissues, which are less accessible in mouse. Rabbits are also phylogenetically well-positioned, sharing a more recent common ancestor to both rodents and primates than other mammalian model organisms, such as the cow or pig^14^. Finally, compared with mice, phylogenetic models predict a smaller branch length from the Glire ancestor, suggesting that the rabbit genome is also more representative of the ancestral condition^15,16^. Despite these advantages, a deep molecular characterisation of early development in the rabbit, and a thorough molecular comparison of rabbit to humans and mouse is lacking.

In this paper, we characterize rabbit development via high-resolution whole-embryo histology, single-cell transcriptomics and single-cell chromatin accessibility profiling, at three gestational days (GDs) 7-9 that capture implantation, amniogenesis, and gastrulation. Using a neighborhood-based approach, we place the rabbit transcriptional landscape in context with the mouse, identifying conserved and divergent cell states, cell types and developmental trajectories. We also investigate the molecular programmes underlying trophoblast differentiation and yolk sac hematopoiesis in the rabbit. Furthermore, we use the fine-grained cell type annotations of both rabbit and mouse atlases to obtain a more detailed view into the cell type diversity of sparse human and macaque data. These comprehensive imaging and single-cell ‘omic resources can be explored via an interactive website available at https://marionilab.github.io/RabbitGastrulation2022/.

## Results

### A time-resolved atlas of rabbit gastrulation and organogenesis

**Figure 1:**
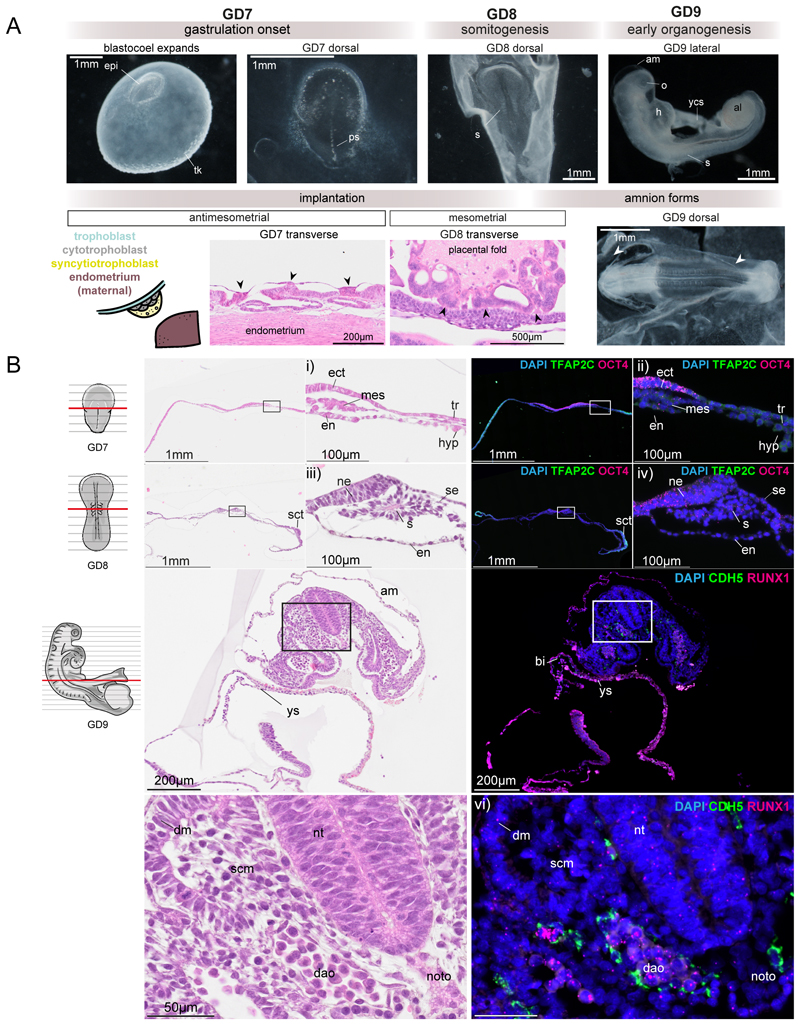
Rabbit timeline of development and high-resolution imaging series of rabbit embryos. **A)** Timeline of developmental milestones in rabbit development using representative images of sampled embryos (see Supplementary Table 1, 2 for sample collection details), and histology images at GD 7, 8, and 9. **B)** Representative images of adjacent serial sections stained with hematoxylin-eosin (H&E) providing morphological information adjacent to specific gene expression detection using RNAscope. Boxes indicate 40x magnification and details shown in i)-vi). i) H&E image of GD7 and ii) adjacent section with RNAscope image of *TFAP2C* and *OCT4* that distinguish ectoderm and trophoblast. iii) transverse section of early somite stage GD8 embryo and iv) *TFAP2C* mark trophoblast and nonneural ectoderm and *OCT4* can be seen lowly expressed in neuroectoderm (ne). v) somite derivatives, neural tube and dorsal aortae can be visualized in H&E. vi) *RUNX1* and *CDH5* visualization of blood cells and endothelium in the dorsal aortae. noto, nototchord; nt, neural tube; sct, syncytiotrophoblast; dao, dorsal aorta; ys, yolk sac; epi, epiblast; ect, ectoderm; mes, mesoderm; end, endoderm; tk, trophoblastic knobs; ps, primitive streak; s, somites; se, surface ectoderm; am, amnion; ch, chorion; o, optic vesicle; h, heart; ycs, yolk connecting stalk; al, allantois; bi, blood island; scm, sclerotome; dm, dermomyotome.

Up until the blastocyst stage, rabbit and mouse embryos share a largely similar structure with an outer layer of trophectoderm surrounding epiblast and hypoblast cell layers^17^. However, in contrast to the mouse embryo, which implants around day 4.5^18^, the rabbit implants during gastrulation after the blastocyst has filled with fluid and expanded. GD 7, 8 and 9 of New Zealand White Rabbit development encompasses implantation, gastrulation and early organogenesis. At GD7, the expanded blastocyst and the first signs of implantation are observed on the anti-mesometrial side of the embryo (Figure 1A, Extended Data Figure 1A)^19^. Additionally, the primitive streak can clearly be seen and the flat-disc-shaped gastrula is similar to the shape of a CS7 human gastrula^20^. By GD8, amnion formation and the first somites can be seen^21^. This is the stage of development at which implantation completes on the mesometrial side, near the embryo-proper (Figure 1A-B, Extended Data Figure 1B). By GD9, the earliest structures corresponding to more mature organs can be observed, such as the optic vesicle, heart, neural tube, and allantois (Figure 1A, Extended Data Figure 1C). Individual embryos across each of these stages were processed to obtain an anatomical and morphological view of rabbit embryogenesis via high-resolution imaging on serial sections with alternating hematoxylin-eosin and RNAScope in situ hybridization staining. This histological reference features over 650 rabbit 4-6µm sections with a section interval as fine as 20 µm (Methods, Figures 1A-B, Extended Data Figures 1A-E). These images include embryos sectioned in-utero and finely sectioned ex-utero embryos. The RNAscope images are performed on some adjacent serial sections, allowing for the combination of morphology and in situ information (Figure 1B). High-resolution RNAscope can also visualize clusters of RUNX1+ CDH5+ haemogenic endothelial cells in the ventral side of the emerging dorsal aorta in the rabbit, suggesting that an early wave of hemogenic endothelium may foreshadow the subsequent development of haematopoietic stem cells in this anatomical location (Extended Data Figure 1E). The largest clusters can be seen close to the entry to the vitelline artery (VA), reminiscent of observations in humans from later developmental stages^22^.

**Figure 2:**
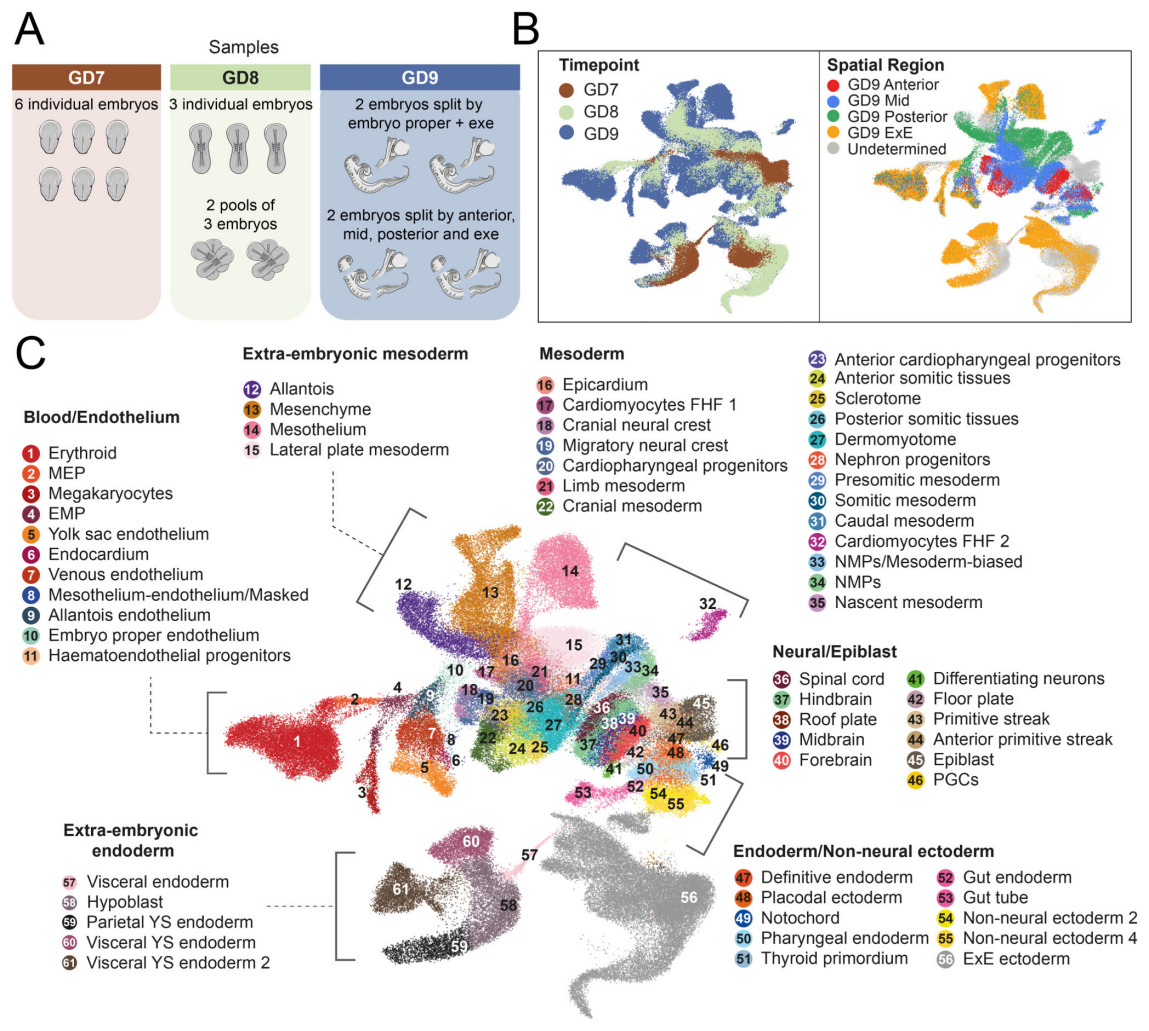
Rabbit transcriptional landscape of gastrulation and early organogenesis. **A)**, An illustration summarizing the samples processed for single-cell RNA-sequencing. **B)** UMAP of 146,133 cells captured in a transcriptional atlas, colored by (left) developmental stage and (right) anatomical region based on the microdissection of GD9 embryos. **C)** The same UMAP in B, labeled by annotated cell type. Each UMAP visualizes cells integrated across all samples (N=19 embryos). UMAP coordinates and cell annotations are available in the Source Data. MEP = megakaryocyte–erythroid progenitors; EMP = erythro-myeloid progenitors; FHF = first heart field; NMPs = neuromesodermal progenitors; PGCs = primordial germ cells; ExE = extraembryonic; YS = yolk sac.

In conjunction with reporting comprehensive histology information, we molecularly profiled cells of the rabbit embryo across the same developmental stages. 6 individual embryos for GD7, 3 individual embryos and 2 pools of 3 embryos each for GD8, and 4 embryos for GD9 were processed for single-cell RNA-seq using the 10X Genomics Chromium System (Methods, Figure 2A). All GD9 embryos were dissected into the embryo-proper and extraembryonic region; two of which were micro-dissected further into the anterior, mid, and posterior regions, providing high-level spatial information (Figure 2B, Extended Data Figure 1G). Following quality control and initial processing, including substantial improvement of the rabbit transcriptome annotation (Methods, Extended Data Figure 2), we obtained high-quality transcriptome profiles for 13,674 cells at GD7, 34,686 cells at GD8 and 97,773 cells at GD9, with a median sequencing depth of 10,126 UMIs/cell (Figure 2C, Extended Data Figure 3).

To assign each profiled cell to a specific cell type, we employed a combination of automated and manual annotation approaches. Using a recent atlas of mouse gastrulation and early organogenesis^23^, we trained a label-transfer model on the full mouse dataset and used this to predict cell type annotations for the rabbit (Methods, Extended Data Figures 4A,B). The preliminary annotations obtained from this automated stage were subsequently verified by manual annotation via assessment of known cell type markers and by cross-referencing with additional information such as the developmental stage, histology, spatial information via embryo microdissection (Extended Data Figure 1), RNA-scope images and independent data integration (Extended Data Figure 4C, see Methods). In total, we defined 67 cell types across a library of 146,133 cells that encompass epiblast, hypoblast and trophoblast lineages (Figure 2C).

### Gene-regulatory dynamics of trophoblast differentiation in vivo

From the fertilized zygote, the first fate decision is between the inner cell mass (ICM) and the trophectoderm. The trophectoderm can be separated into the top, polar trophoblast layer overlying the ICM, and the bottom mural trophoblast of the blastocyst. In species such as rabbit, dog, sheep, and pig, the polar trophoblast layer degenerates so that the epiblast becomes contiguous with the mural trophoblast^24^. The remaining trophoblast cells subsequently differentiate to cytotrophoblast and syncytiotrophoblast, which mediate implantation and restructuring of the maternal environment to accommodate the developing embryo.

Despite being of vital importance for the successful development of the embryo, the gene-regulatory programmes underlying trophoblast differentiation and the establishment of the fetal-maternal interface in vivo remain poorly understood. In humans and mice, implantation takes place very early in development , with consequent challenges in capturing enough cells for rigorous molecular profiling. Moreover, in organisms where the embryo is deeply embedded within the uterine lining (such as human and mouse), capturing trophoblast cell types without also capturing a lot of maternal material is experimentally challenging. By contrast, in the rabbit, implantation occurs alongside gastrulation at GD 7 and 8^3,19^, when the embryo is larger, enabling the straightforward capture of large numbers of extraembryonic-ectoderm cells (Figure 1A, 2C).

**Figure 3:**
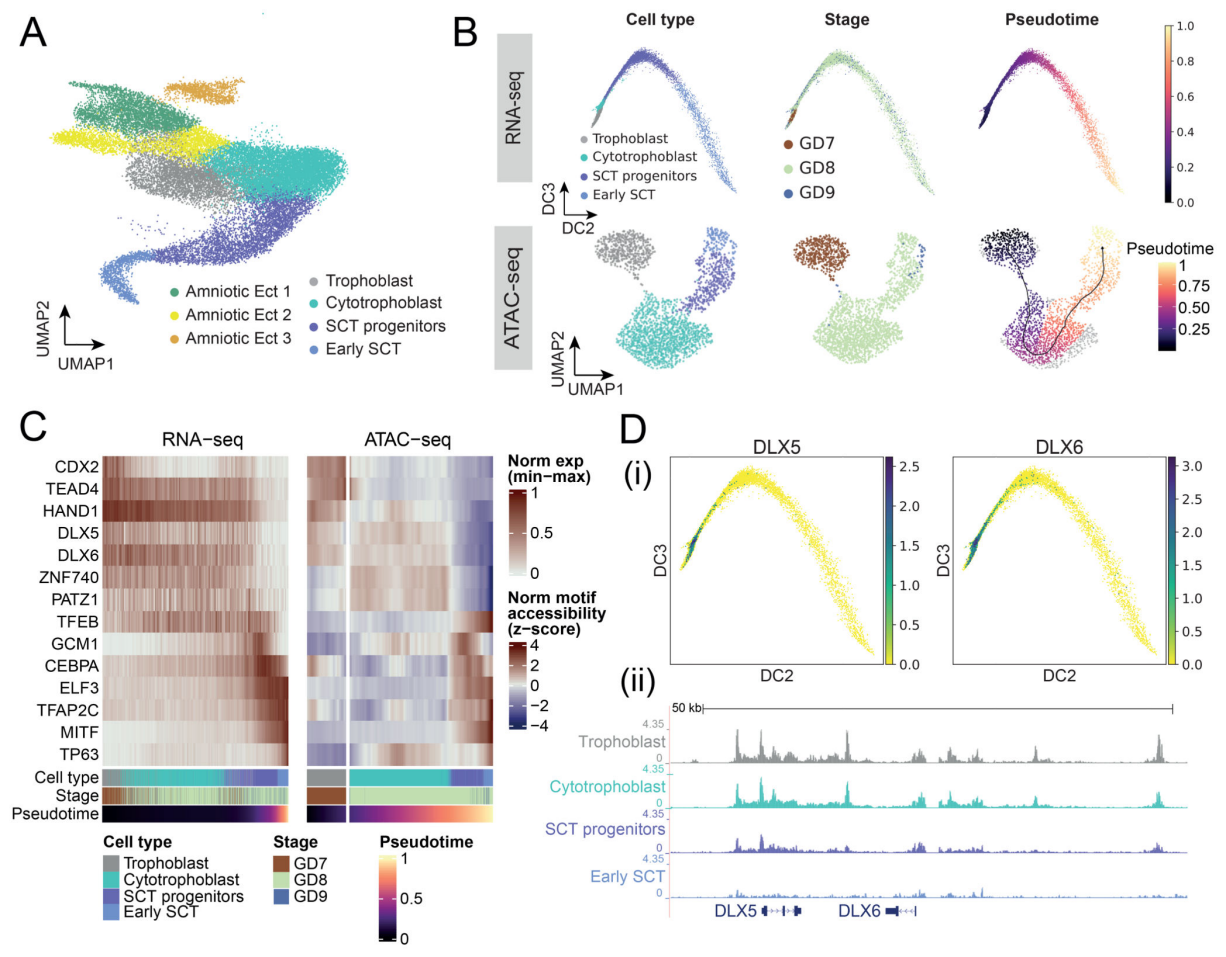
Chromatin accessibility and gene expression along the trajectory of early syncytiotrophoblast differentiation **A)** Refined cell type annotation of extra-embryonic ectoderm cells presented in the UMAP embedding (n=26,853 cells). SCT; syncytiotrophoblast. **B)** Trophoblast cells/nuclei of the RNA-seq (top, n=19,291 cells) and ATAC-seq (bottom, n=2,386 nuclei) datasets are represented in low-dimensional embeddings which highlight a trajectory towards early syncytiotrophoblast. Cells are coloured by cell type (left), developmental time point (middle) and pseudotime (right), calculated independently for the RNA-seq and ATAC-seq atlases using diffusion pseudotime and ArchR trajectory inference respectively. The RNA-seq data is plotted with respect to the second and third diffusion components, whereas the ATAC-seq data is represented in a UMAP embedding. **C)** Heatmap of smoothed TF expression (left) and motif enrichment (right) along cells/nuclei of the RNA-seq and ATAC-seq SCT trajectories, ordered by their respective pseudotimes. The cell type, stage, and pseudotime value for each cell is indicated below. **D)** i) Expression of DLX5/DLX6 is restricted to trophoblast and cytotrophoblast cells, shown in the diffusion map embedding (n=19,291 cells). ii) Genome browser view of the region surrounding the DLX5/6 locus, showing downregulation of accessibility along the differentiation trajectory. All dimensionality reduction plots represent cells integrated across all samples (RNA: 19 embryos; ATAC: 4 embryos) where those cell types are present. Normalized expression values, motif accessibility scores, dimensionality reduction coordinates and cell-based annotations are available in the Source Data.

Consistent with this, in comparison to the mouse gastrulation dataset ^4^ which only captures a subset of cells labeled as extraembryonic-ectoderm, we are able to profile a diverse set of extraembryonic ectoderm cell types (Figure 3A, Extended Data Figure 5D), encompassing the maturation of early trophoblast cells into cytotrophoblast and finally into syncytiotrophoblast (SCT) progenitors. Multinucleated syncytium were not captured due to their invasion of the maternal layer during dissection.

To complement the transcriptional profiling of different trophoblast cell-types, and especially given the relatively limited information about trophoblast development in vivo, we performed single-cell assay for transposase-accessible chromatin-sequencing (scATAC-seq) across GD7-9, resulting in chromatin accessibility profiles of 34,082 cells after quality control (Extended Data Figure 6; Supplementary Table 2). Using the archR pipeline^25^, cells were clustered and gene accessibility scores were used to perform cell type label transfer from the transcriptional atlas. Clusters containing cells of the different extraembryonic-ectoderm cell types were re-analysed and the cell type annotation was manually curated by inspection of chromatin accessibility of key marker genes and enrichment of transcription factors (TFs) motifs.

Ordering both the scRNA-seq and scATAC-seq data of the trophoblast-syncytiotrophoblast trajectory along pseudotime (Figure 3B) allowed us to compare the differential TF motif accessibility of known and putative regulators of trophoblast development and correlate this with differential expression of the matched scRNA-seq data (Figure 3C). Consistent with trophoblast development in human and mouse, we see enrichment for the binding motifs of TEAD4^26^ and CDX2^27^ at regions accessible during early trophoblast timepoints, which rapidly close as trophoblast cells mature. In cells assigned a cytotrophoblast identity, we observed accessibility in regions associated with DLX5/6, ZNF740, and TP63 motifs. Later, during syncytiotrophoblast differentiation, we observed open chromatin in regions associated with the GCM1 motif ^28^, a known regulator of CEBPA and syncytin genes, the latter of which are directly involved in cell-cell fusion with the maternal decidua^29,30^. Finally, in the most mature syncytiotrophoblast cells, motifs associated with the binding of TFEB and MITF are highly accessible. These TFs are known to form homodimers or heterodimers and are involved in vascularization and placental labyrinthine development^31^. Overall, these patterns are consistent with the limited information available for human, not only suggesting conservation of key temporal changes in chromatin accessibility during trophoblast development, but also establishing a comprehensive in vivo dataset capturing this key process at single-cell resolution.

Interestingly, some patterns of changes in chromatin accessibility seen in the rabbit do not conform with previous mouse data; for example, we observed changes in chromatin accessibility at motifs associated with DLX5/DLX6 binding during cytotrophoblast differentiation, as well as corresponding expression (Figure 3D). These genes are known to be expressed in humans but are not expressed in murine trophoblast^32^. DLX5 and DLX6 are also known preeclampsia markers, with 69% of preeclamptic placentas showing upregulation in one study^32^. Moreover, these genes are implicated in proliferation of trophoblast and are normally downregulated during the differentiation process to syncytiotrophoblast. This conservation of markers between rabbits and humans suggests that the rabbit may be a useful model system for examining genes linked to preeclampsia.

### A neighborhood-based comparison of cell states across species

**Figure 4:**
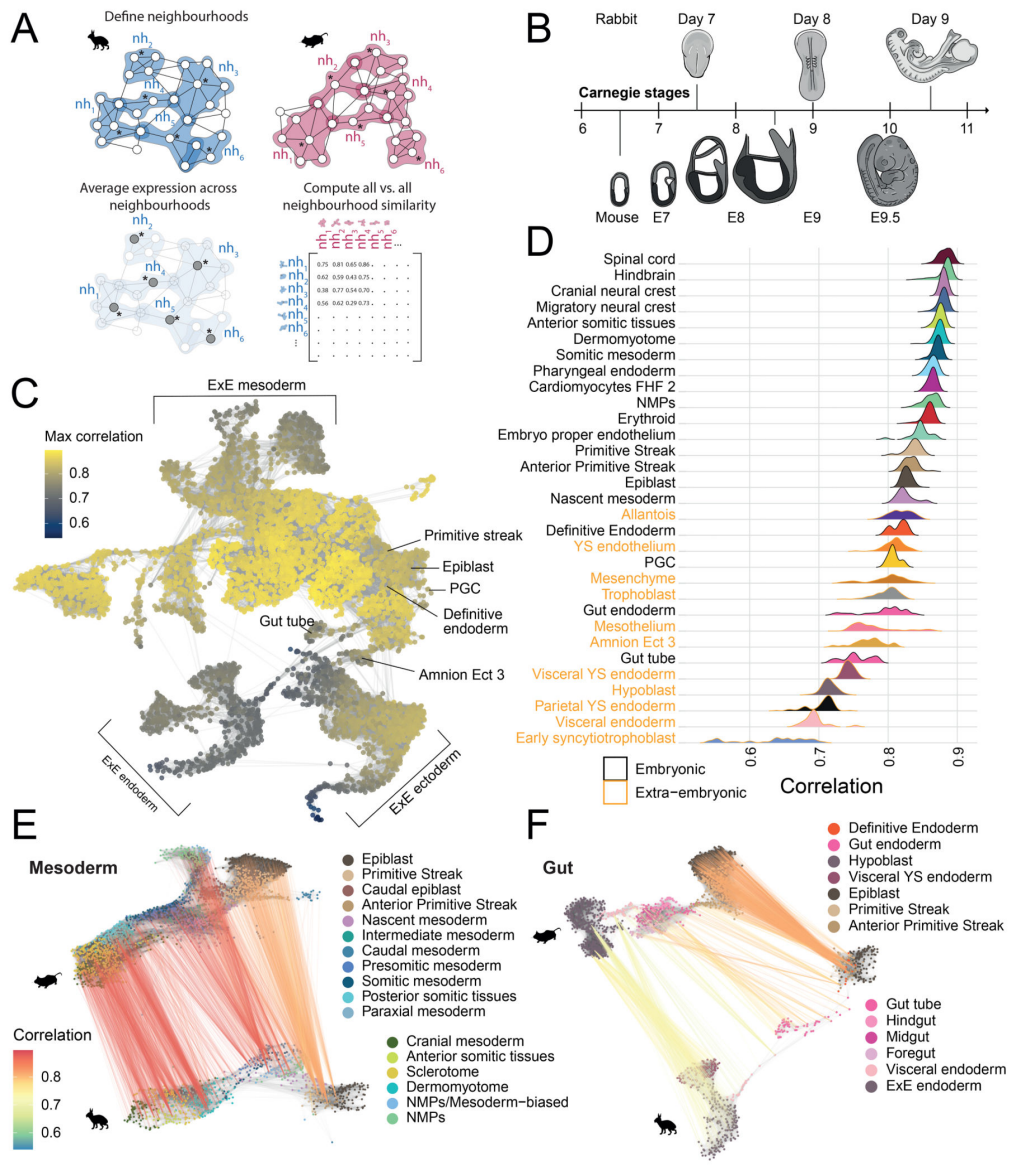
Rabbit and mouse neighborhood comparisons identify regions of similarity and dissimilarity across species. **A)** Schematic of our neighborhood comparison approach. Index cells (marked by *) are sampled across the kNN graphs of each species. The k-nearest neighbors of each index cell then collectively define overlapping neighborhoods. The average expression profile within each neighborhood is calculated and the correlation in expression across all neighborhoods of both species is represented in a matrix of neighborhood similarities. **B**) The timepoints sampled in both the rabbit and mouse ^23^ atlases are related according to Carnegie staging. **C)** Rabbit neighborhoods, positioned with respect to the UMAP embedding of each index cell (n=5,253 neighborhoods), are coloured by the maximum correlation value across all mouse neighborhoods, highlighting regions of higher and lower similarity with the mouse. The underlying kNN graph is shown in grey. **D)** Maximum correlation scores are aggregated by the cell type of each neighborhood index cell. The distribution of maximum correlation scores are shown, for a selected subset of cell types, in a ranked ridgeline plot (ordered by mean maximum correlation). Extra-embryonic cell types are highlighted in orange. See a complete list in Extended Data Figure 6B. **E, F)** A subset of rabbit and mouse neighborhoods associated with mesoderm (**E)** and gut (**F**) differentiation trajectories are shown. Maximally correlated rabbit and mouse neighborhood pairs (computed in both directions) are connected, where the line colour represents the value of maximum correlation. In panels C-F, the rabbit neighborhoods are constructed from cells integrated across all samples (N=19 embryos). Maximum correlation values and mappings between neighborhoods are available in the Source Data.

An embryo-wide comparison of transcriptional states, between the rabbit and mouse, would help to elucidate which developmental processes are conserved across species and which show divergence. To do this, we developed a computational approach to evaluate transcriptional similarity between k-nearest neighbor (kNN) graphs, constructed from scRNA-seq data of different species (Figure 4A). Independently for each species, we defined neighborhoods of cells across the entire kNN graph^33^ allowing us to locally approximate the transcriptional profile in precise regions of the gene expression manifold. In contrast to cell types or discrete clusters, neighborhoods are typically more granular, are independent of cell type annotations, and more optimally encapsulate gene expression changes, especially across continuous trajectories. Having defined neighborhoods within each species, we compute an average expression profile for each neighborhood before computing a correlation matrix (using orthologous genes) to measure the similarity between all pairs of neighborhoods across species (Methods).

We applied this approach to compare our transcriptional atlas of rabbit development with the E6.5 - E9.5 single-cell atlas of mouse development used in our cell type annotation^23^. These timepoints overlap with our GD7, GD8 and GD9 rabbit samples, as assessed by Carnegie staging (Figure 4B). Using the matrix of correlations, computed across 5,253 rabbit and 14,034 mouse neighborhoods (Extended Data Figure 7A), we first investigated which regions of the rabbit developmental landscape were conserved or divergent with the mouse. To do this, for each rabbit neighborhood, we extracted its maximum correlation value across all mouse neighborhoods (Figure 4C).

The most similar neighborhoods between species correspond to cell types within the embryo proper, particularly within the anterior and mid sections of the embryo (Extended Data Figure 7D), including the neural crest, nervous system and mesodermal cell types (Figure 4D, Extended Data Figure 7B). By contrast, we noted that neighborhoods representing extraembryonic cell types, such as the amnion, parietal and visceral yolk sac endoderm, were among the least correlated cell types with the mouse, possibly reflective of the known morphological differences and divergent modes of development. For instance, the amnion forms from a single amnio-chorionic fold in mice that grows into the proamniotic cavity^34^, while, in rabbits, both anterior and posterior folds converge following degeneration of Rauber’s layer (Figure 1A). Similarly, the visceral yolk sac is much larger in rabbits and encloses the embryo much later in development^35^. Additionally, several embryonic cell types show relatively high divergence including the gut, primordial germ cells (PGCs) and cell types of the early GD7 embryo (Figure 4C,D; Extended Data Figures 7B,D). Interestingly, in the mouse, extraembryonic cell types play critical roles in the development of both the gut and the PGCs, via cell intercalation and signaling, respectively^36,37^. In the former context, embryonic definitive endoderm and extraembryonic visceral endoderm intercalate to form the early gut tube. Previous studies have shown that these intercalated gut endoderm cells retain a transcriptional signature of their embryonic and extraembryonic origin^38^, indicating that differences in extraembryonic tissues may persist in cells of embryonic tissues. The specification of PGCs in mice is also reliant on signaling from the polar trophoblast, which degenerates in rabbits^37,39^. The rabbit embryo exhibits a flat-disc at GD7, which may also explain the low correlation values at this earliest time point.

To investigate these observations in more detail, we examined how similarities between neighborhoods vary along trajectories of differentiation. Specifically, we visualized mappings between maximally correlated neighborhoods along the reduced dimensional spaces of rabbit and mouse datasets for a subset of cell types relating to the development of specific lineages (Figure 4E-F, Extended Data Figures 8A-C). While neighborhoods map very strongly across the whole spectrum of mesodermal cell types (Figure 4E), the trajectory of endoderm development is much less correlated between species (Figure 4F). As in Figure 4D, the extraembryonic component of the gut trajectory exhibits the lowest similarity between the rabbit and mouse, whereas neighborhoods representing the epiblast and definitive endoderm, are more strongly correlated. Moreover, many neighborhoods of the rabbit gut tube at GD9, have highest correlation with mouse neighborhoods at E8, suggesting a change in the timing of development (Extended Data Figure 8D).

Taken together, our neighborhood-based analysis provides a general approach to compare single-cell RNA-seq datasets at a high level of granularity and independently of cell type annotations. Applying this to the rabbit and mouse atlases we observe differences in extraembryonic tissues and in cell types such as the gut and PGCs, whose development is known to be influenced by extraembryonic structures and are linked to the maternal environment and cup vs disc embryo morphology^40,41^. These results have implications for how we interpret observations from specific cell types and lineages in any one species and set the scene for more extensive cross-species analysis when suitable datasets are reported for additional species.

### Rabbit atlas expands understanding of early primate development

The quantity of cells, tissues and developmental stages captured in the rabbit and mouse atlases provide a comprehensive view into the development of these organisms. Given the scarcity of transcriptomic data from primate embryos, we investigated whether the comprehensive mouse and rabbit resources could be leveraged, using automated cell type annotation tools, to gain deeper insight into the cellular makeup of sparse human and macaque datasets. Manually annotating cell types de-novo can be a challenging and time-consuming process, particularly when cell types are represented by small numbers of cells. In these cases, there is often little statistical power to confidently detect signals in marker gene expression above transcriptional noise. Overcoming these difficulties using model organism reference atlases will become increasingly relevant as more studies of early human development take place. To take advantage of our newly generated rabbit data, as well as the existing mouse datasets, we used SingleR to train cell type annotation models on the rabbit and mouse atlases. We then used these models to predict cell type annotations from the transcriptomic profiles of a representative human and macaque single-cell query dataset.

**Figure 5:**
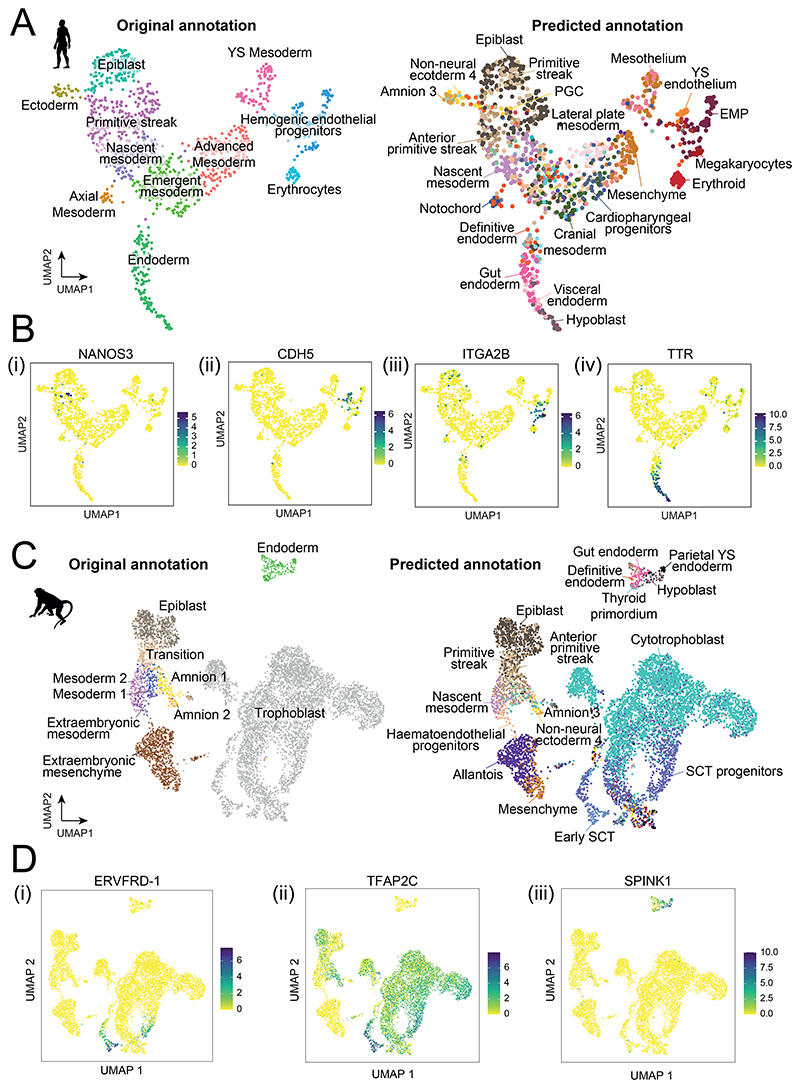
Automated annotation models trained on the rabbit atlas accurately classify cell types in sparse human and macaque data. **A)** UMAP of the Tyser et al. 2021 human embryo SMART-seq v2 data (1,195 cells, N=1 embryo) coloured according to the original cell type annotations (left) and the cell type labels predicted from training a SingleR model on the rabbit atlas (right). **B**) The rabbit model predictions are consistent with the expression patterns of known i) PGC, ii) endothelium iii) megakaryocyte and iv) extra-embryonic endoderm marker genes (1,195 cells, N=1 embryo). **C)** A UMAP of macaque scRNA-seq data from Yang et al. 2021 (7,194 cells, N=12 embryos), coloured according to the original (left) and predicted (right) cell type annotations. **D**) i) Expression of the macaque syncytin gene, ERVFRD-1, and ii) TFAP2C, validate the model predictions of early syncytiotrophoblast and syncytiotrophoblast progenitors. iii) Predictions of hypoblast and parietal YS endoderm also overlap with the expression of extra-embryonic endoderm marker, SPINK1 (7,194 cells, N=12 embryos). The UMAPs in panels C-D depict cells integrated across all samples. Normalized expression values, predicted cell type annotations and UMAP coordinates are available in the Source Data.

Tyser et al. 2021 reported a SMART-seq v2 dataset of 1,195 cells, obtained from a single CS7 human embryo between 16 and 19 days post-fertilisation^20^. At this stage of development, the human embryo appears as a flat-disc, similarly to the rabbit embryo at day 7. Based on the transcriptomic profiles of these cells, we observe that our rabbit annotation model assigns several additional cell type annotations to those consistent with the original publication (Figure 5A, Extended Data Figure 9A). For instance, cells originally classified as ‘hemogenic endothelial progenitors’ are subclassified into yolk sac endothelium, erythroid myeloid progenitors (EMP) and megakaryocytes (Figure 5A), which we were able to validate using known marker genes (Figure 5B). The model also identifies amniotic ectoderm cells and two PGCs. In the original study, these cell types were identified via a refined subclustering, requiring both complex computational analysis and a high-degree of domain expertise. Interestingly, the mouse-trained annotation model failed to classify these PGCs (Extended Data Figure 9B). Given the relatively low neighborhood similarity scores between rabbit and mouse PGC neighborhoods, this suggests that the transcriptomic profile of rabbit PGCs are closer to that of the human. This is consistent with a recent study of PGCs in the rabbit which suggests that key regulators of PGC specification are shared across flat-disc species^42^. Other differences are found in the prediction of epiblast cells, which the mouse-trained model labeled as ectoderm.

We applied the same approach to a single-cell RNA-sequencing dataset of cultured in-vitro macaque embryos spanning embryonic days 10, 12 and 14^43^. These stages of development correspond with Carnegie stages 5-6^44^ reflecting an earlier developmental stage than captured in our rabbit dataset, although partially overlapping with the E6.5-E7.5 stages of the mouse. Despite this, we find that both mouse and rabbit annotation models are able to replicate and refine the major cell types annotated in the original publication (Figure 5C, Extended Data Figures 9C,D). In addition to the epiblast, primitive streak, nascent mesoderm and amnion cells, which are concordant with the original labels, both rabbit and mouse-trained models separate cells of the ‘extraembryonic mesenchyme’ cluster into allantois and mesenchyme annotations and distinguish gut endoderm cells from parietal and extraembryonic endoderm. Furthermore, the rabbit model predicts two domains of syncytiotrophoblast and syncytiotrophoblast progenitors within the original trophoblast labeled cluster. These regions overlap with the expression of the macaque-unique syncytin gene, *ERVFRD-1*, and *TFAP2C* (Figure 5Di,ii). Given that implantation occurs around day 9.5-10.5 in the macaque^45^, immediately prior to the timepoints represented here, it is possible that the trophoblast is transitioning through a similar differentiation process as observed in our rabbit atlas at GD8.

The ability of the mouse and rabbit references to precisely and accurately annotate distinct cell types within the human and macaque datasets illustrates the utility of using a more comprehensive reference set when performing cell type annotation. While the rabbit-trained model more accurately identified human PGCs and epiblast cells, the mouse-trained model predicted a smoother transition of mesodermal cell types across the UMAP embedding, possibly reflective of the higher resolution of 6-hour timepoints sampled (Extended Data Figure 9B). This emphasizes the strengths of using both atlases, in other studies of mammalian embryogenesis.

### Rabbit Blood Niche Mirrors Human Blood Culturing Conditions

In the previous sections we showed that the rabbit is an especially advantageous model system for studying extra-embryonic tissues. The yolk sac is an important site for haematopoiesis and early fetal nutrition when the chorioallantoic stalk is not yet formed. Amongst these cell types, our transcriptomic atlas contained a large number of yolk sac mesothelium cells, which have not been studied in great detail before. Additionally, we capture cells of the yolk sac endoderm, blood cells, and yolk sac endothelium. The large size of the rabbit yolk sac also makes it easy to image, dissect, and characterize. Given the key role the yolk sac plays in early development, we leverage imaging and computational analysis to infer cell-cell communication between adjacent cell types.

**Figure 6:**
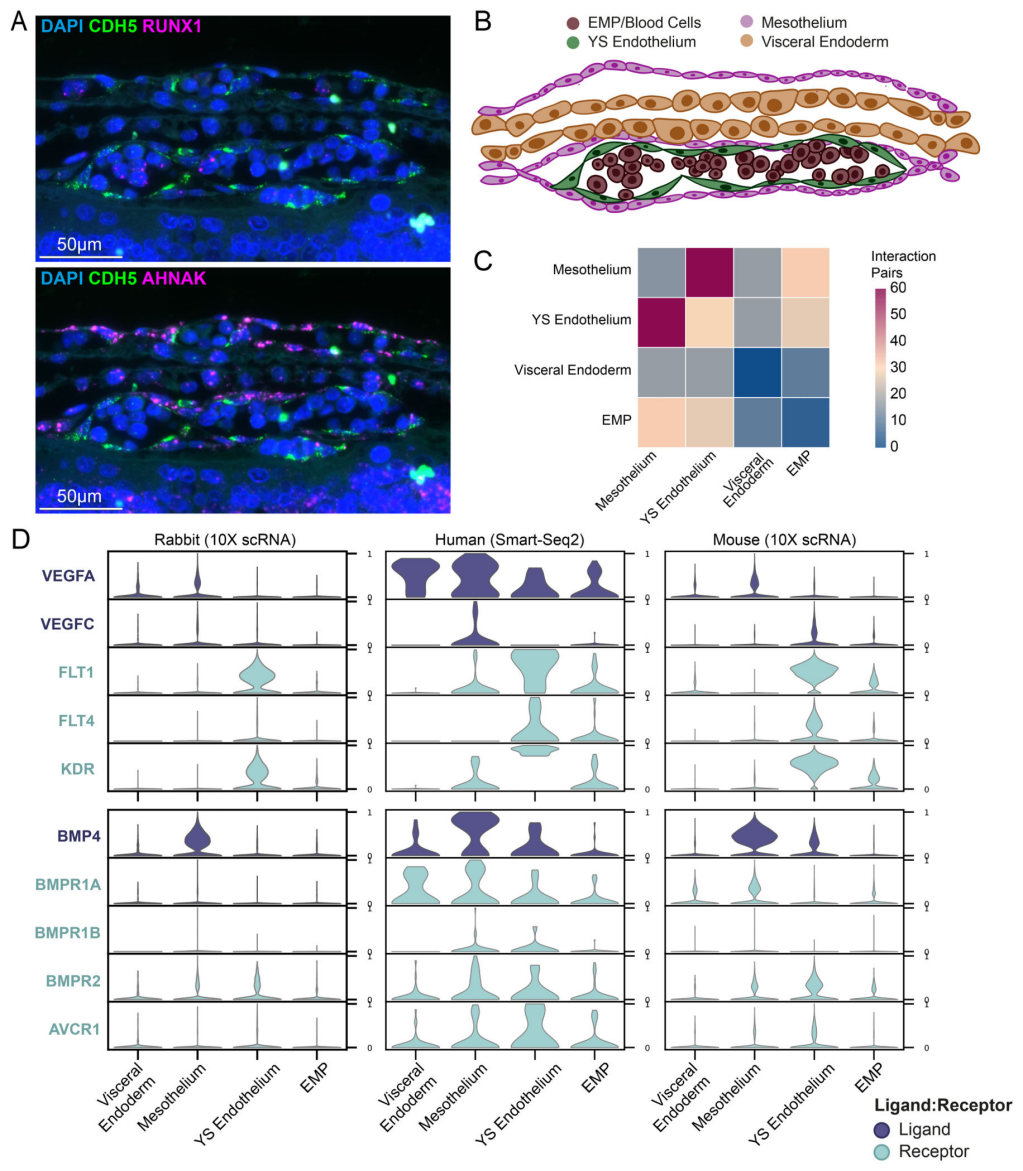
Rabbit yolk sack haematopoiesis exhibits conserved molecular markers with human in vitro development models and reveals signaling role of mesothelium **A)** RNAscope image of yolk sac hematopoiesis with DAPI nuclear staining with probes for CDH5 and RUNX1 expression (left) and CDH5 and AHNAK expression (right). Cells are distinguished with RUNX1^+^ blood, CDH5^+^ endothelium, AHNAK^+^ yolk sac mesothelium and AHNAK^-^/CDH5^-^ visceral yolk sac endoderm. **B**) Schematic of the rabbit yolk sac haematopoietic niche according to the RNAscope images in A). **C)** Heatmap of CellPhoneDB interaction pair counts across different cells located within the yolk sac blood islands. **D)** Violin plots of ligand-receptor expression across the yolk sac blood island cells for signaling involved in haematopoiesis such as VEGF/VEGFR (FLT1, KDR), and BMP/BMPR across the rabbit transcriptional atlas (left), CS7 human gastrula (Tyser et al. 2021, middle), and extended mouse transcriptional atlas (Imaz-Rosshandler et al. 2023, right). The ligands are colored in dark blue-purple, while the receptors are colored in cyan. Plots in panels C and D (left) were generated using cells integrated across all samples (N=19 embryos) where yolk sac haematopoetic cell types were present. Expression values and interaction counts are available in the Source Data.

To this end, we mined our sc-RNAseq dataset for marker genes that distinguish the different layers of the yolk sac hematopoietic niche in rabbit at GD9, and spatially resolved their localisation in situ, using RNAscope imaging. With spatially resolved RNAscope experiments, we identified blood cells, which can be seen inside of vessels formed by *CDH5*^+^ endothelium (Figure 6A). Some of the blood cells are *RUNX1*^-^ while others are *RUNX1*^+^. *AHNAK*^*high*^ mesothelium cells can be seen surrounding these *CDH5*+ endothelium cells (Figure 6A), with the blood cells in the center. The layer of large *AHNAK*^low^ endodermal cells matching the morphology of mouse endoderm cells can be seen opposite the *AHNAK*^high^ layer. Visual inspection of these layers revealed that this basic structure commonly occurs in pairs, with the endoderm layers apposed to each other to form a mirrored bilayer structure constituting the rabbit blood islands (Figure 6B).

The mesothelium has been relatively overlooked as a potential signaling center for the maturation of blood cells in the yolk sac compared to the more studied yolk sac endoderm, thought to provide inductive signaling to the YS endothelium^46,47^. By leveraging the resolution of RNAscope, we observed that *AHNAK*^+^ mesothelium cells are in direct contact with the YS endothelium. We also used CellPhoneDB^48^ to predict the ligand-receptor interactions for these adjacent cell types (Extended Data Figure 10A). After subsetting the rabbit dataset to include cells associated with the yolk sac blood islands, we observed that the highest score of inferred interaction pairs are between mesothelium and YS endothelium (Figure 6C). Gene ontology analysis reveals that the visceral yolk sac endoderm scores highly on cholesterol efflux and transport, indicative of its role in transporting and metabolizing nutrients^49^ (Extended Data Figure 10B), with the highest scoring gene functions related to extracellular matrix and structure. Putative interactions between mesothelium and YS endothelium include various extracellular matrix proteins like nectin, fibronectin-1 (FN1), collagen genes and important blood maturation ligands like *BMP4, VEGFA* and *VEGFC*.

Leveraging the refined mesothelium annotation of the human CS7 dataset (Figure 5A) we compare expression across the rabbit, mouse and human. This analysis revealed high expression of *VEGFA/C* from the mesothelium and endoderm, BMP4 signalling from mesothelium, and higher receptor expression in the YS endothelium across the three species (Figure 6D)^50^. Interestingly, the addition of VEGF was previously shown to promote in vitro primitive blood maturation from human pluripotent cells, yet the in vivo source of the cytokine had remained a mystery^51^. Given previous reports on BMP4-mediated induction of haematopoietic development^51^, these results highlight a likely role for mesothelium in the yolk sac hematopoietic niche across mammalian species in supplementing signaling from the endoderm.

## Discussion

We report high-resolution morphological and molecular maps of early rabbit development, covering GD 7, 8 and 9. Using cutting-edge single-cell genomics approaches, we characterized the transcriptional and chromatin accessibility profiles of over 180,000 individual cells, isolated from whole rabbit embryos, across gastrulation and early organogenesis.

Our results highlight the utility of the rabbit as a model for a new wave of mammalian development research that will combine the power of traditional comparative embryology with state-of-the-art comparative single-cell genomics approaches. When researching rabbit development for this manuscript, one useful resource was the 1905 ‘Plate’ of rabbit development, published as part of a compilation of 16 “Normal Plates of the Development of the Vertebrates”, edited by the German anatomist Franz Keibel (16 volumes, 1897–1938)^52^. It may have taken over 100 years, but it is now realistic to imagine a similar compendium of single-cell genomics atlases for over a dozen vertebrates within the next few years. The ability to query expression of drug targets, such as key receptors and signaling pathways, across these multi-organism atlases will ultimately lead to improved knowledge of species-specific drug responses and teratogenic effects. Coupled with innovation in experimental techniques such as embryo culture, genome modification and lineage tracking, single-cell comparative genomics will then likely emerge as a new energizing force accelerating the use of model organisms to decipher early human development and drive advances in translational medicine.

## Methods

All experimental procedures on rabbits were undertaken by Covance and were subject to the provisions of the United Kingdom Animals (Scientific Procedures) Act 1986 Amendment Regulations 2012 (the Act). The number of animals used was the minimum that is consistent with scientific integrity and regulatory acceptability, consideration having been given to the welfare of individual animals in terms of the number and extent of procedures to be carried out on each animal.

### Histology

Timed mating: Male/female ratio 1:1. The female and male mated twice (morning and afternoon). Post-mating, the female rabbit was injected intravenously with luteinising hormone (dose 25 i.u., Luveris (75 IE, Merck)). The day of mating was counted as GD 0. Pregnant rabbits from timed matings were euthanized at GD 7, 8 and 9 by an i.v. administration of a lethal dose of pentobarbital (0.35 ml/kg, Euthasol (400 mg, Dechra Veterinary Products A/S)). The uteri were dissected out and transferred to ice-cold phosphate buffered saline. Some embryos were dissected out while others were left intact in uterus to preserve extraembryonic structures. Dissected tissues were transferred to 10% neutral buffered formalin then allowed to fixate for 48 to 96 hours. After fixation the specimens were processed in an automated tissue processor (Leica ASP300S) and embedded in paraffin.

The paraffin embedded specimens were serially sectioned on an AS-410M fully automatic microtome (Axlab) producing 6µm sections spanning the entire embryo in each specimen or serially sectioned manually at 4µm on a Leica RM2255 microtome and collected on Superfrost Plus slides (Epredia, J7800AMNZ). Slides were deparaffinized with xylene and rehydrated to water. Mayer’s hematoxilin solution (Sigma-Aldrich, MHS80) was applied for 5 minutes followed by washing in tap water for 5 minutes. Eosin (Sigma-Aldrich, HT110280) was then applied for 5 minutes followed by washing and dehydration in a graded ethanol series to xylene. The slides were mounted with pertex and whole slide scanned on an Olympus VS200 scanner using a 20x NA 0.8 objective, 40x NA 0.95 objective, and 40x NA 1.4 oil objectives (Olympus, UPLXAPO40XO).

### RNAscope

mRNAs were detected using the automated RNAscope LS Multiplex Fluorescent Assay on a Leica Bond RX autostainer (probe sets available on request). Slides were deparaffinized (Leica Biosystems, AR9222), pretreated with BOND Epitope Retrieval Solution 2 (Leica Biosystems, AR9640) and protease (ACD, 322800) followed by probe hybridization. Probes were detected using the RNAscope LS Multiplex Reagent Kit (ACD, Cat. No. 322800) and Opal 520, Opal 570 and Opal 690 fluorophores (Akoya Biosciences, 1487001KT, FP1488001KT, FP1497001KT). Slides were counterstained with DAPI (Sigma-Aldrich, D9564), mounted with Prolong Diamond antifade mountant (Thermo Fisher Scientific, P36970) and whole slide imaged on an Olympus VS200 slide scanner equipped with DAPI, FITC, Cy3 and Cy5 filter sets using 20x NA 0.8, 40x NA 0.95 objective, or 40x NA 1.4 oil objectives. Images were processed for publication using Olympus OlyVIA.

### Rabbit Embryo processing for scRNA-Seq

New Zealand White Rabbits (Oryctolagus cuniculus) from Envigo (RMS) UK Ltd, were mated at Labcorp Early Development Laboratories Limited (Eye, Suffolk; formerly known as Covance Laboratories Limited) by natural mating. After mating, each female was injected intravenously with 25 i.u. luteinizing hormone. GD0 was defined as the day of mating. On GD7, 8 or 9 the pregnant rabbits were sacrificed, and uteri were harvested and shipped fresh in Phosphate Buffered Saline (PBS) on ice to the Jeffrey Cheah Biomedical Centre. Embryos were dissected using a Leica brightfield microscope and fine point tweezers using 10% heat-inactivated Fetal Bovine Serum (FBS) in PBS. Embryos were selected based on morphology matching the developmental day of dissection as described previously^52^. Selected embryos or dissected structures (see Supp Table) were rinsed in PBS, centrifuged at 100 x g for 3 minutes before being individually dissociated with TrypLE™ Express (Gibco™) by incubating for 6-10 minutes at 37°C with occasional agitation of the tube by flicking its base to ensure even dissociation of the embryo. Dissociation was quenched with 5mL 10% heat-inactivated FBS in PBS and filtered using a 30µm Sysmex CellTrics® filter. Cells were centrifuged at 300 x g for 3 minutes and resuspended in 0.04% BSA in PBS. Cells were filtered through a 40μm Flowmi tip strainer (VWR) to minimize volume loss during filtration and then counted using a haemocytometer.

Six GD7 embryos were dissociated separately, and processed as individual samples. Two pools of 3 GD8 embryos with no visible somites were processed, as well as 3 GD8 individually processed embryos with 4 somites apiece. Two GD9 embryos were split between embryo proper and extraembryonic tissues. Embryo proper portions were dissociated and split across two separate samples. Another 2 GD9 embryos were individually processed and split into the anterior, mid, posterior, and yolk-sac region. Cell solutions and scRNA-seq libraries were processed by the CRUKCI genomics core facility using Single Cell Gene Expression v3 from 10X Genomics following manufacturer’s instructions. Samples were sequenced following manufacturer’s recommendations on an Illumina NovaSeq 6000 platform.

### Rabbit embryo processing for scATAC

Embryos dissected from the same time-points for transcriptional profiling were flash frozen by placing whole embryos into Corning® Cryogenic Vials and immediately submerging the vials in liquid nitrogen. Embryos were stored at -80°C. Nuclei were extracted following a 10X Genomics protocol for single cell ATAC on frozen tissues (CG000212). Two GD7 embryos were pooled for nuclei extraction, 1 GD8 embryo was split across two samples, and 1 GD9 embryo was split into the extraembryonic portion for one sample and the embryo portion was split across four samples. Frozen embryos were placed on ice with 500uL chilled 0.1X lysis buffer and homogenized using RNase-Free Disposable Pellet Pestles (Fisher Scientific). Samples were filtered using a 70 µm Flowmi Cell Strainer followed by a 40 µm Flowmi Cell Strainer. Nuclei were counted in a haemocytometer using Trypan Blue Solution 0.4% (Sigma-Aldrich, Cat. No. T8154-20ML) and were resuspended in 1X Nuclei Buffer (10X Genomics). Nuclei solutions and libraries were processed by the CRUKCI genomics core facility using Single Cell ATAC (v1.1) from 10X Genomics following manufacturer’s instructions. Samples were sequenced following manufacturer’s recommendations on an Illumina NovaSeq 6000 platform.

### Improving transcriptome mapping

After processing the single-cell RNA-sequencing data with Cell Ranger using the Ensembl OryCun2.0 rabbit reference transcriptome, we observed a low percentage of reads mapping to the transcriptome. Visualizing read coverage, we discovered that a large majority of the 10X sequencing reads were aligning to regions upstream of the 3’ end of annotated genes (Extended Data Figure 2A-C). Furthermore, several genes that are known to be well-conserved, were missing from the rabbit annotation. To rectify this, we extended the 3’ annotation of genes by 600bp and added human genes that aligned to unannotated regions in the rabbit genome.

The decision to extend genes by 600bp was determined by analyzing distances of intergenic reads from their nearest annotated gene (Figure S2A). Intergenic reads were extracted from the Cell Ranger BAM output file of the SIGAC11 GD8 (Supplementary table 1) sample by filtering on the ‘RE’ BAM alignment tag using SAMtools. This revealed that the majority of reads fell within 600bp from the 3’ end of the nearest gene, suggesting that a 600bp extension would provide a reasonable compromise between capturing missing reads and avoiding overlaps with nearby annotations. We also prevented this explicitly, ensuring that extensions were only added to each gene providing they did not overlap with an existing annotation. The GTF file of the rabbit transcriptome was modified by adding CDS and exon entries to ensure they were counted by the Cell Ranger pipeline.

To obtain a list of features that may be absent from the rabbit annotation, we identified genes that have a one-to-one orthology relationship between the mouse and human but were missing from the rabbit reference. Given the human gene ID for each feature, we located positions in the human genome relating to transcripts of that gene and specifically, the positions of each transcript’s 3’ most exon and UTR. Using the Ensembl Compara Perl API, we then obtained alignments for these sequences in the rabbit transcriptome, taking only those with maximum alignment scores. Exon and CDS annotations spanning these positions were then added to the rabbit GTF file, similar to the extended 3’ ends. Additional measures were taken to ensure that the alignments extracted did not overlap existing annotations (including the added extensions) and that alignment sequences for the same transcript were proximal (i.e. not mapping to different chromosomes).

As a result of these changes, the median number of genes and UMIs per cell increased from 1875 to 2661 and from 6987 to 10,126 respectively. Moreover, 1648 additional genes were added, including several known marker genes such as *FOXC1* and *SIX3* (Extended Data Figure 2C-E).

### scRNA-seq preprocessing via Cell Ranger

FASTQ sequencing files were processed with Cell Ranger 3.1.0 using default settings. Reads were mapped to the OryCun2.0 genome from Ensembl with the modified 3’ extension GTF annotation file mentioned previously.

### Quality control for scRNA-seq

Swapped barcodes for molecular counts were corrected as previously described (Pijuan-Sala et al. 2019) by applying DropletUtils (version 1.6.1) function ‘swappedDrops’ (default parameters) to groups of samples that were multiplexed for sequencing. Cell calling and doublet removal was performed as described previously (Pijuan-Sala et al. 2019). See the Github repository for more details: https://github.com/MarioniLab/RabbitGastrulation2022. Cells with considerably lower mitochondrial gene expression and smaller total UMI counts compared with other clusters were removed as previously described (Pijuan-Sala et al. 2019), likely corresponding to clusters of nuclei stripped of their cytoplasm.

### Normalization for scRNA-Seq

Transcriptome size factors were calculated as previously described (Pijuan-Sala et al. 2019) using ‘computeSumFactors’ from the scran R package (version 1.18.7). See the Github repository for more details: https://github.com/MarioniLab/RabbitGastrulation2022.

### Batch correction for scRNA-Seq

Batch effects were removed using the ‘fastMNN’ function from the batchelor R package (version 1.6.3). The top 3000 HVGs were calculated with the ‘modelGeneVar’ and ‘getTopHVGs’ functions in ‘scran’. These were used by fastMNN to compute the top 50 principal components. We enforced a particular order in which to combine samples, using the ‘merge.order’ parameter of ‘fastMNN’, merging samples in reverse order of developmental stage and in decreasing order of the number of cells within each timepoint. For the GD9 samples, we merged samples of the same anatomical dissection before those from different parts of the embryo. All 26 samples were integrated into a combined principal component space, with these principal components used for all downstream analysis steps.

### Visualization and clustering

A UMAP embedding of the whole dataset was computed using Scanpy version 1.9.1 (‘scanpy.api.tl.umap’) ^53^. The 300 nearest neighbors in the batch-corrected principal component analysis were considered, with a ‘min.dist’ parameter of 0.9. Force-directed graph layouts were computed on the batch corrected PCs with the ‘scanpy.api.tl.draw_graph’ function using the ForceAtlas2 algorithm.

The whole dataset was clustered using the Leiden algorithm (scanpy.api.tl.leiden) using the same neighborhood graph constructed for the UMAP embedding. Clusters were generated using a range of resolution parameters (1 - 10) to assist in making cell type annotations at varying levels of coarseness. We also performed clustering within known lineages and subsets of the data to capture different sources of variation.

### Cell type annotation

Cell type annotations were initially predicted by training an automated annotation model on a recent single-cell atlas of mouse gastrulation and early organogenesis ^23^. This dataset consists of 430,339 cells across embryonic days 6.5 to 9.5, sampled at 6-hour intervals, which were manually curated into 87 different cell types. To transfer annotations from the mouse to the rabbit, we utilized SingleR (version 1.4.1), an automated annotation method that assigns cell type labels based on correlation in expression between reference and query cells ^54^. To relate the rabbit and mouse datasets, we constructed a common feature set between the atlases using one-to-one ortholog genes. We then trained a SingleR model on the mouse atlas (with ‘trainSingleR’ from the ‘SingleR’ package using parameters ‘de.n = 50’ and ‘de.method = Wilcox’), providing the cell type annotations from the original study, using the pseudobulk option within SingleR.

Since the annotation model makes predictions independently for each query cell, clusters with a dominant cell type prediction were suggestive of a reliable annotation (Extended Data Figure 4A, B).

47/67 cell types defined in the rabbit were annotated consistently with the mouse (Supplementary Table 3). Based on evidence from marker gene expression, automated cell type annotation and SAMap integration, these cell types presented a 1-1 mapping with those in the mouse (Extended Data Figure 4A,C). Cell types were annotated differently between the two datasets if they were i) not confidently identifiable in the rabbit or mouse, ii) if a different terminology was preferred or iii) if a less-specific annotation was more suitable (Supplementary Tables 4-7).

5/67 cell types fell into this last category - particularly neural cell types. Forebrain, midbrain and hindbrain populations were detectable in the rabbit atlas, based on signatures of expression (Forebrain: OTX2+, FEZF1/2+, SIX6+; Midbrain: OTX2+, FEZF2-, HOXA2-; Hinbrain: HOXA2+, HOXA7-, HOXB9-, OTX2-). However, the mouse forebrain was subclassified into 'Ventral forebrain progenitors', 'Late dorsal forebrain progenitors' and 'Early dorsal forebrain progenitors', which are not as clearly defined in our rabbit data. Similarly the mouse atlas distinguishes 'Midbrain progenitors' from 'Dorsal midbrain neurons' and specifies a 'Midbrain/Hindbrain boundary'. These separations were inconspicuous in the rabbit atlas and so a lower resolution classification was used. The same was true of the 'Hindbrain' (subclassified into 'Hindbrain neural progenitors', 'Ventral hindbrain progenitors' and 'Dorsal hindbrain progenitors' in the mouse) and 'Spinal cord' (annotated as 'Dorsal spinal cord progenitors' and 'Spinal cord progenitors' in the mouse). Mouse 'Branchial arch neural crest' and 'Frontonasal mesenchyme' were also grouped into a 'Cranial neural crest' cell type in the rabbit. The automated label transfer of these cell types did not form distinct clusters in the rabbit, although they were proximally located in the UMAP embedding (Extended Data Figure Figure S4A) and aligned closely with cells from anterior GD9 samples (Figure 2B).

5/67 cell types were annotated inconsistently due to different choices of terminology. For example, the rabbit cluster of 'Differentiating neurons' exhibited strong similarity with 'Dorsal midbrain neurons' of the mouse (Extended Data Figure 4A,C), however, there was no strong evidence indicating the spatial specificity of these cells in the rabbit, suggesting that a less-specific annotation was more suitable. These cells differentially express markers of early neural differentiation (NEUROD4 and ASCL1). The spatial specificity of the rabbit 'Floor plate' (SHH+) was also indiscernible and so was labelled more generally than the 'Hindbrain floor plate' annotation in the mouse. The neighbourhood comparisons and SAMap integration suggest that the rabbit rA3 cluster is closely related with the mouse 'Non-neural ectoderm 3' cell type (Extended Data Figures 4A, 4C, 8B). However these cells express markers of the amniotic ectoderm, such a GABRP and VTCN1^21^, and so this cluster was annotated as 'Amnion 3' in the rabbit. 'Trophoblast' and 'Hypoblast' cell types were also renamed from 'ExE ectoderm' and 'ExE endoderm' respectively to reflect the uncertain relationship between extra-embryonic tissues and match terminology more commonly used in non-rodent embryology.

Finally, 10/67 cell types, were not present in the mouse atlas and were annotated de-novo in the rabbit. This mostly includes extra-embryonic ectoderm cell types (Amnion 1, Amnion 2, Cytotrophoblast, SCT progenitors, Early SCT). Genes used to annotate these clusters are shown in Extended Data Figure 5D. Concretely, the amnion 1 cluster was annotated based on the expression of markers associated with the first-wave of amniogenesis^21^. This includes VTCN1, CDX2 ^21^ and TFAP2C^43^, as well as aquaporin and prostaglandin genes, additionally implicated in amniotic fluid homeostasis ^55^. Other amnion markers, KRT7 and WNT6 ^56^ were used to annotate Amnion 2. The third amnion cluster, Amnion 3 specifically exhibited strong similarity with surface-ectoderm derived amnionitic ectoderm and the second-wave of amniongenesis, expressing GABRB, ISL1 and WNT6^21^. These genes have been reported in the mouse, macaque and human amniotic ectoderm ^21,43,56,57^. Markers of the trophoectoderm, such as HAND1, CDX2^58^ and TEAD4^59^ were used to annotate the Trophoblast which also primarily originates from the earliest GD7 timepoint (Figure 2B). The cytotrophoblast, consisting mostly of GD8 cells, was annotated based on a similar transcriptional signature (TEAD4+, 'HAND1+, WNT2,) as well as the expression of FGFR2^60^ and CYP19A1, which is upregulated during cytotrophoblast-syncytiotrophoblast differentiation ^61^. Synctiotrophoblast progenitors were identified through the expression of GCM1 and CEBPA, key regulators of synctial fusion ^62,63^. Finally the early synctiotrophoblast population was annotated based on the expression of TFAP2A/C and TP63, which are similarly detected in human and mouse syncytiotrophoblast ^64,65^. In addition to the extra-embryonic ectoderm clusters, the roof plate was identifiable as a distinct neural cluster that differentially expressed LMX1A^66^. A definitive endoderm annotation was also absent from the mouse atlas and so this was annotated based on the expression of GSC, EOMES and FOXA2^67^. While the definitive endoderm was straightforward to detect, the relationship between more differenitated rabbit and mouse endoderm cell types was less clear, specifically cell types of the gut. The automated label transfer model annotated a trajectory of FOXA2+ cells as the ‘Midgut’ (Extended Data Figure 4A). However, while the mouse defines foregut, midgut and hindgut cell types, these subdivisions are not identifiable in the rabbit and are potentially associated with later developmental stages (Extended Data Figure 8A). Hence the GD9 subset of predicted gut cells (DKK1+, PLAT+) were annotated as the 'Gut tube', (consistently with the mouse), while those predominately at the earlier GD8 timepoint was labelled as 'Gut endoderm’. The gut tube annotations of the rabbit and mouse overlap in the SAMap embedding (Extended Data Figure 4C). ‘Visceral YS endoderm’ and ‘Visceral YS endoderm 2’ clusters were also given unique labels as their relationship with the mouse was not clear. These cells express markers of the extra-embryonic visceral endoderm, such as APOA2, CITED1 and TTR^38^.

There was insufficient evidence to annotate the additional 26/87 cell types present in the mouse atlas.

Cell type colours were chosen to most easily facilitate comparisons between similar cell types in the mouse. For 52/67 annotations, where there is a confident or suspected 1-1 mapping between cell types, consistent colours was used. Additional colours were chosen with consideration to visual clarity and palettes used for similar tissues.

To visualise marker genes enriched in each annotated cell type, the ‘findMarkers’ function was used from the scran R package (version 1.18.7), with parameters ‘direction = up’, and ‘pval.type=some’. Extended Data Figure 5B, shows min-max normalised expression (across each gene) of one differentially expressed gene per cell type.

### Rabbit-mouse integration

The rabbit and mouse ^23^ datasets were integrated into a joint embedding using SAMap version 0.1.6 (Extended Data Figure 4C) ^68^. An initial mapping between rabbit and mouse features was first obtained by reciprocal BLAST aligning the rabbit and mouse transcriptomes. The transcriptomes were obtained through Ensembl and aligned using the ‘map_genes.sh’ script, provided with SAMap (https://github.com/atarashansky/SAMap/). This script outputs a table of sequence similarity scores between rabbit and mouse transcripts, which are used by SAMap as an initial weighting between features. Since the features of the rabbit and mouse datasets are given in terms of Ensembl gene IDs, prior to running SAMap, we linked each Ensembl transcript ID with its associated gene ID. The SAMap algorithm also utilises self-assembling manifolds 83 to align datasets and so these were computed for the rabbit and mouse atlases using Scanpy (scanpy.tl.external.sam). We then constructed a SAMAP object, providing the rabbit and mouse SAM objects, the transcript to gene mappings and the map_genes output directory. We then ran the SAMap pipeline on this SAMAP object (SAMAP.run) to integrate the two datasets. For visualisation purposes, we re-computed a UMAP embedding on the integrated anndata object (SAMAP.adata) using a minimum distance parameter of 0.8.

### RNA-seq trophoblast analysis

Cells annotated as trophoblast, cytotrophoblast, syncytiotrophoblast progenitors and early syncytiotrophoblast were isolated for trajectory analysis. A diffusion map low-dimensional embedding^69,70^ was computed (using ‘scanpy.api..tl.diffmap’) with 30 components on the batch corrected kNN graph. The GD7 trophoblast cell with highest value in its second diffusion component was then chosen as the root cell for computing diffusion pseudotime^71^ (with scanpy.api.tl.dpt) on the 30 diffusion components, specifying 0 branchings.

In Figure 3C, cells were ordered according to diffusion pseudotime. Expression values for each gene were then smoothed along this pseudotemporal ordering by calculating a moving average with a window size of 49 cells. These smoothed values were normalised to between 0 and 1 using min-max scaling. The same procedure was used to plot the ATAC-seq motif accessibility values, except that cells were ordered along pseudotime calculated in ArchR (see ‘scATAC-seq trophoblast analysis’) and a z-score normalisation was applied in replace of min-max scaling, to account for positive and negative accessibility scores. The combined heatmap was plotted using the ‘ComplexHeatmap’ package (version 2.6.2) in R.

### scATAC-seq pre-processing

FASTQ files of scATAC-sequencing were mapped to the rabbit genome (OryCun2.0) using cellranger-ATAC version 2.0.0. Full analysis is performed using the ArchR pipeline (version 1.0.1)^25^, where reads on unplaced scaffolds in the rabbit genome were ignored in the pipeline. Arrow files were created from CellRangers fragment files using createArrowFiles, using minTSS=2 and minFrags=3000, followed by doublet detection and removal using addDoubletScores with k=15. After inspecting quality distributions, new thresholds were determined (minTSS=2.9 and minFrags=8000). A total of 34,082 cells passing quality control were used in the analysis (Supplementary Table 2, Extended Data Figure 6).

### scATAC-seq dimensionality reduction and peak calling

The TileMatrix, containing read counts per 500 bp bins of the entire genome, was used for a first round of dimensionality reduction using addIterativeLSI, using dimsToUse=1:45 and nFeatures=60000 and clustering using addClusters with resolution=1. These clusters were then used to create ‘Pseudo-Bulk Replicates’ using addGroupCoverages with useLabels set to TRUE. These Pseudo-Bulk Replicates were subsequently used for peak calling with Macs2 ^72^ (version 2.2.7.1) using addReproduciblePeakSet, with genomeSize=2.7e9, cutOff=0.001, and extendSummits=250, resulting in a final set of 332,773 peaks. The detected peaks were then used to create the PeakMatrix, containing read counts per peaks. The second round of dimensionality reduction and clustering was performed on the PeakMatrix, using the same parameters as for the TileMatrix. UMAP was performed with addUMAP, using nNeighbors=45 and minDist=0.5.

### scATAC-seq and scRNA-seq integration

To perform cell-type annotation of the scATAC-seq cells, integration was performed with the labeled scRNA-seq dataset for each stage separately. Dimensionality reduction was performed using addIterativeLSI, using dimsToUse=1:35 and nFeatures=55000 and clustering was performed using addClusters with resolution=1.5 to create high-resolution per stage clusters. Imputation matrices were added using addImputeWeights. Next, scRNA-seq integration was performed using addGeneIntegrationMatrix, which compares the estimated gene activity stored in the ‘GeneScoreMatrix’ for scATAC-seq with the gene expression of the scRNA-seq dataset in order to perform label transfer for cell types and add a ‘GeneIntegrationMatrix’ containing integrated RNA expression counts.

### scATAC-seq label transfer verification

Pseudobulked gene scores per cell-type were calculated from the scATAC-seq gene score matrix by generating a SingleCellExperiment object and running scuttle (v1.4) aggregateAcrossCells with ids = label transfer cell types and statistic=‘mean’. Pseudobulked transcriptomes per cell-type profiles were generated from the scRNA-seq by aggregateAcrossCells with ids = annotated cell types and statistic=‘sum’, with subsequent normalization by dividing each value by the total reads per cell-type, multiplied by 1e6, and log2 normalized. Cell types with fewer than 20 cells in scATAC-seq were removed from analysis. Cell-type specific marker genes were determined by first pseudobulking scRNA-seq by cell-type and sample, followed by scran’s (v1.22) pseudoBulkDGE where each cell-type is tested against all other cell types, with samples given as replicates. Top marker genes were determined by taking those genes with a logFC>1 and FDR<0.05 and taking the top 50 genes with highest logFC for each cell-type. RNA-ATAC comparison was performed by taking the scaled mean expression and GeneScore per cell-type, across the top marker genes for each cell-type separately. Linear regression was then performed to determine the correspondence of ATAC and RNA profiles.

### scATAC-seq motif deviations

For each peak, TF motifs were detected using addMotifAnnotations, searching for motifs in the ‘cispb’ set for Homo Sapiens, as the motifs between human and rabbit are expected to be highly conserved. To compute motif deviations, first a set of background peaks were detected using addBgdPeaks, followed by generation of the ‘MotifMatrix’ by addDeviationsMatrix, which stores the deviation and Z-scores for each motif per cell.

### scATAC-seq trophoblast analysis

Trophoblast and amnion cells were isolated from the total dataset by isolating cells belonging to clusters 1 to 5. To manually curate the annotation of these cells, dimensionality reduction and reclustering was performed with addIterativeLSI, using dimsToUse=1:25 and nFeatures=25000 and addClusters with resolution=1. Markers of the ‘GeneScoreMatrix’ and ‘MotifMatrix’ per cluster were used to relabel Trophoblast, Cytotrophoblast, Syncytiotrophoblast progenitors, Syncytiotrophoblast, and Amnion cells. Next, Amnion cells were excluded and dimensionality reduction was repeated on these cells with dimsToUse=1:10 and nFeatures=15000, and the UMAP was generated using nNeighbors=35 and minDist=0.8. Trajectory analysis was performed by determining pseudotime ordering using addTrajectory over the UMAP embedding. Motifs with variable accessibility across pseudotime were subsetted to those of particular interest, which were correlated with TF expression in the scRNA-seq dataset across trophoblast cell types, or which are known from the literature. The marker gene heatmap was created by taking the mean of the imputed GeneScores for the refined trophoblast annotation using aggregateAcrossCells with statistic=‘mean’, followed by minmax normalization. Motif enrichment was performed by first determining differential regions across trophoblast cell types using ArchR’s getMarkerFeatures, followed by peakAnnoEnrichment with cutOff = "FDR <= 0.05 " Log2FC >= 0.5”, with the heatmap showing a subset of relevant TF motifs.

### Neighborhood comparisons

To define neighborhoods, we used the same construction employed for differential abundance testing ^33^. Independently for the rabbit and mouse, the top 50 PCA components were used to construct a kNN graph (with k=30 neighbors). Neighborhoods were then defined by aggregating the k-nearest neighbours of a randomly sampled set of index cells. The final set of neighborhoods were refined from this initial selection to prevent oversampling and to create larger, more representative neighborhoods (see ^33^ for details). These steps were performed by the `buildGraph` and `makeNhoods` functions from the ‘miloR’ package (version 0.99.19) using a sampling proportion of 0.05. From this we obtained 5,253 rabbit and 14,034 mouse neighborhoods with a mean neighborhood size of 104.8 and 134.4 cells respectively (Extended Data Figure 7A).

We next identified a set of features with which to compare neighborhoods across the two species. Specifically, we selected the intersection of the top 2000 highly variable genes, computed independently for each dataset. Features were selected using the ‘getScranHVGs’ function from the scran R package. We excluded from this mitochondrial genes and those associated with cell cycle GO terms. Finally, we selected only those which are one-to-one orthologs across the rabbit and mouse. Our final set of features consisted of 796 genes.

Using this set, we computed the mean expression profile within each neighborhood (using the normalised and log-transformed gene counts). We also implemented a version of gene-specificity, used in previous cross-species comparisons, to account for differences in quantification and absolute values between datasets ^73,74^. Specifically, the within-neighborhood averages for each gene (*g*_*i*_) were scaled by the mean across all neighborhoods (*i*, … {*1*, ...*N*}).

$$s_{g}^{i}=\frac{g_{i}}{\frac{1}{N}\Sigma_{k=1}^{N}\;g_{k}}$$

The gene specificity values (*S*_*g*_^*i*^) for each gene, across all neighborhoods, were then used to compute the Pearson correlation between all pairs of rabbit and mouse neighborhoods forming a matrix of neighborhood similarity.

### Primate cell type annotation

In our cell type annotation analysis we utilised the Tyser et al. 2021^20^ and Yang et al. 2021 ^43^ single-cell datasets of early human and macaque development. The Tyser et al. 2021 SMART-seq dataset consists of 1,195 cells from a single CS7 human embryo acquired through the Human Developmental Biology Resource. We downloaded the normalised gene expression values and UMAP coordinates from http://human-gastrula.net/. Conversely, the Yang et al. 2020 dataset was generated through 10X genomics single-cell RNA-sequencing of in-vitro cultured macaque embryos, at days 10, 12 and 14. We accessed raw count data through the GEO database under accession number GSE148683 and downloaded associated metadata from the accompanying shiny app at https://www.nhp-embryo.net/. To combine the day 10, 12 and 14 samples, we performed batch correction with fastMNN with k=20. We also normalized and log-transformed the raw counts using ‘computeSumFactors’ and ‘logNormCounts’ functions in the ‘scran’ and ‘scuttle’ R package.

To predict cell types in the human and macaque datasets, we applied the same approach used to annotate the rabbit atlas originally. We trained SingleR annotation models on the rabbit and mouse datasets both jointly and independently. Datasets were filtered to include only one-to-one ortholog genes between each reference (rabbit or mouse) and query (human or macaque) dataset. As before, the training and classification steps were performed using the ‘trainSingleR’ and ‘classifySingleR’ functions from the ‘SingleR’ R package, with the ‘aggr.ref=TRUE’ option. In Figure 5, we display the ‘pruned’ cell type annotations from SingleR, which have undergone a filtering step to remove low-quality assignments.

### CellphoneDB analysis of Yolk Sac Haematopoiesis

Cell-cell interactions were predicted using a previously curated list of ligand-receptor and receptor-receptor pairs using CellphoneDB v.2.0 ^48^. Due to the exponential nature of possible interactions across the full dataset, only a subset of cell types corresponding to the rabbit yolk sac and blood islands were used, covering ‘EMP’, ‘visceral endoderm’, ‘yolk sac endothelium’, and ‘YS mesothelium’. The visceral endoderm cell type in the CellphoneDB heat map analysis includes ‘visceral endoderm’, ‘visceral ys endoderm 1’, and ‘visceral ys endoderm 2’.

### Statistics and reproducibility

Representative microphotographs of rabbit embryos were selected based on the average of morphology as described previously^52^ and of embryos processed (see Supplementary Table 1, 2 for sample collection details). For RNAscope and H&E images, summary of embryos imaged at each time point and total number of sections per time point are summarized in Extended Data Figure 1D. Low quality cells were excluded as detailed in the Methods section. Otherwise, no data from the processed embryos were excluded from the analysis. Statistical details for each analysis can be found in the corresponding subsections of the Methods. Random seeds used in statistical functions are available in the Github repository (see Code Availability). Sample sizes are provided in the figures or figure legends. The experiments were not randomized and the Investigators were not blinded to allocation during experiments or outcome assessment.

## Extended Data

**Figure:**
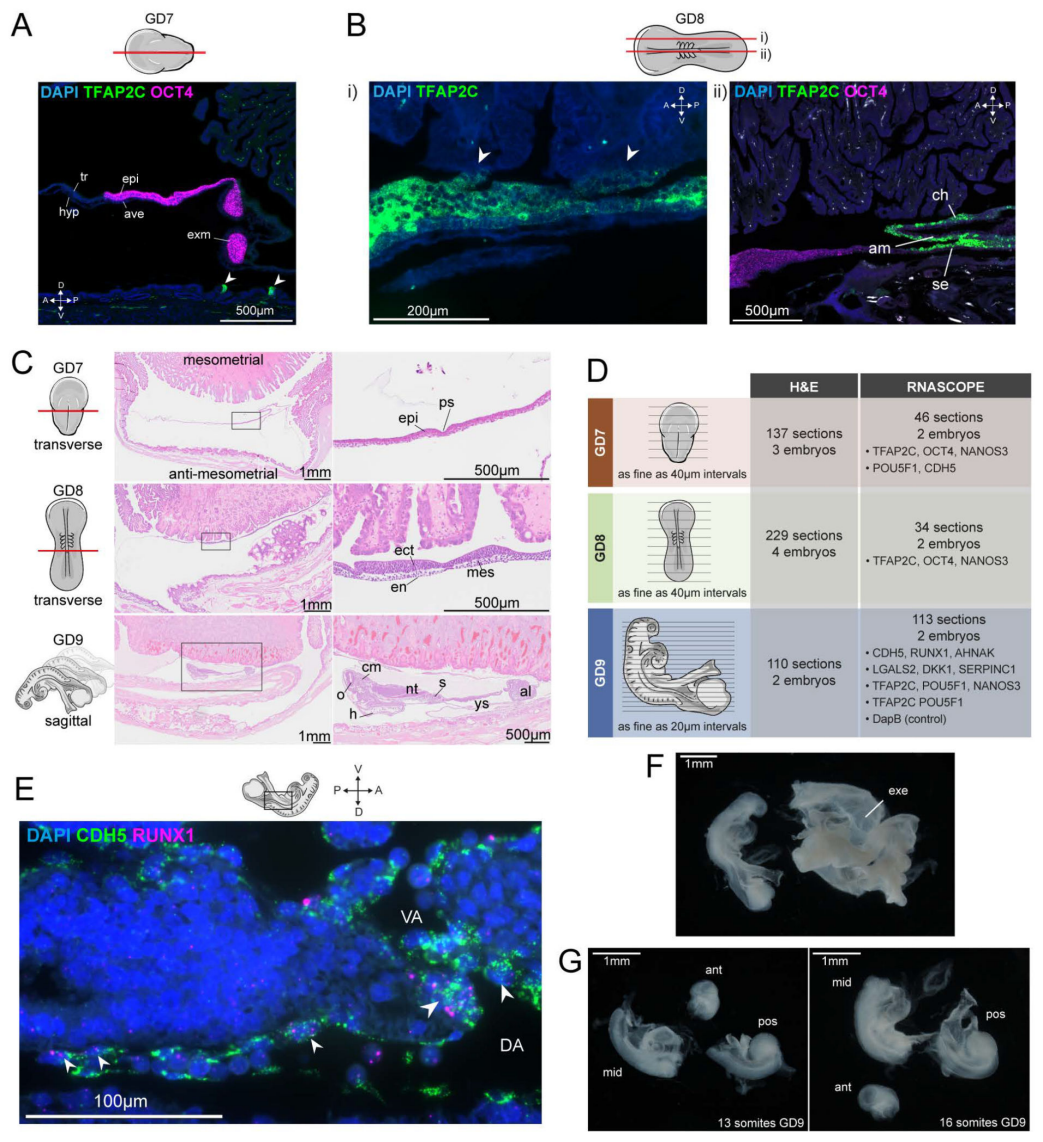


**Figure:**
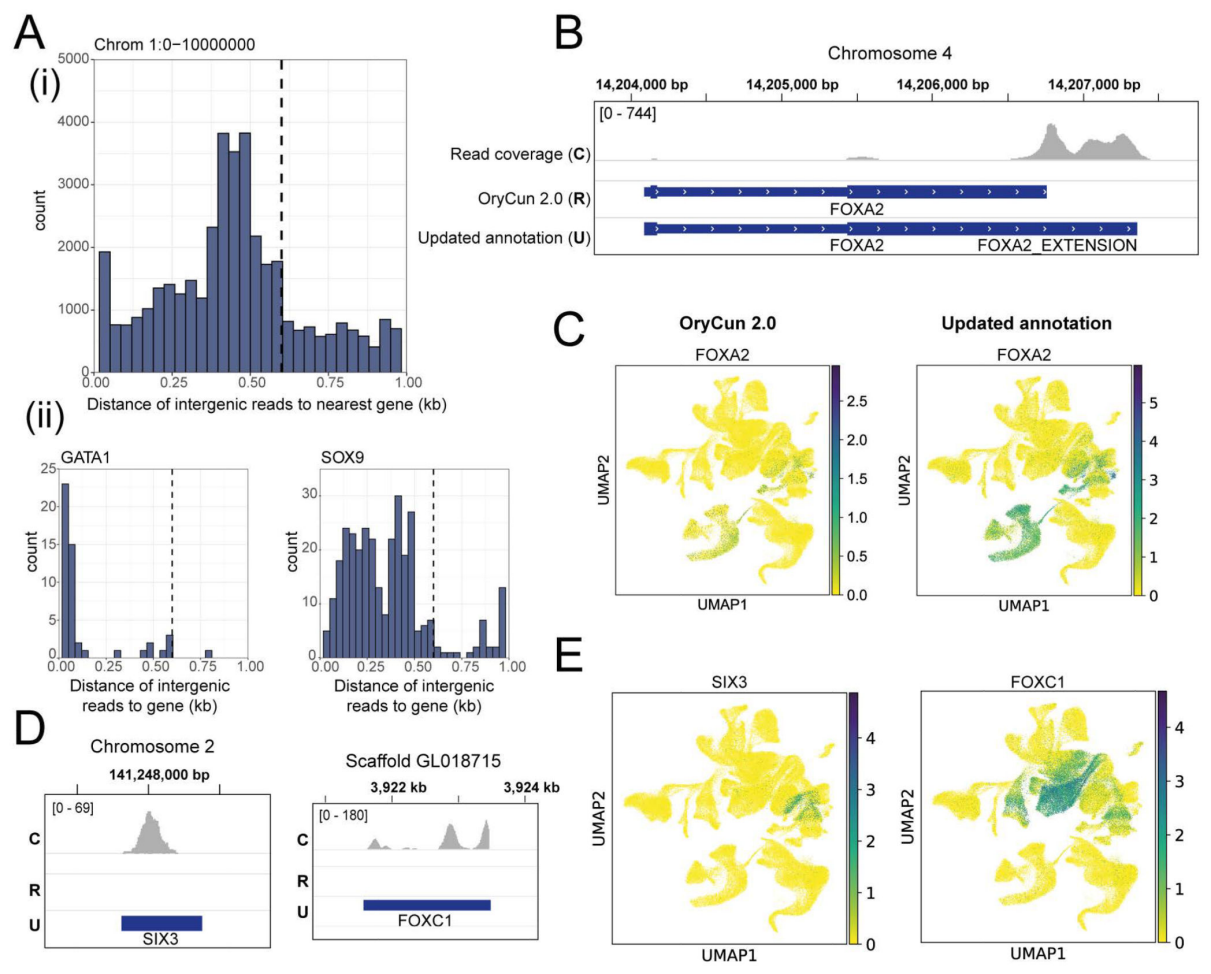


**Figure:**
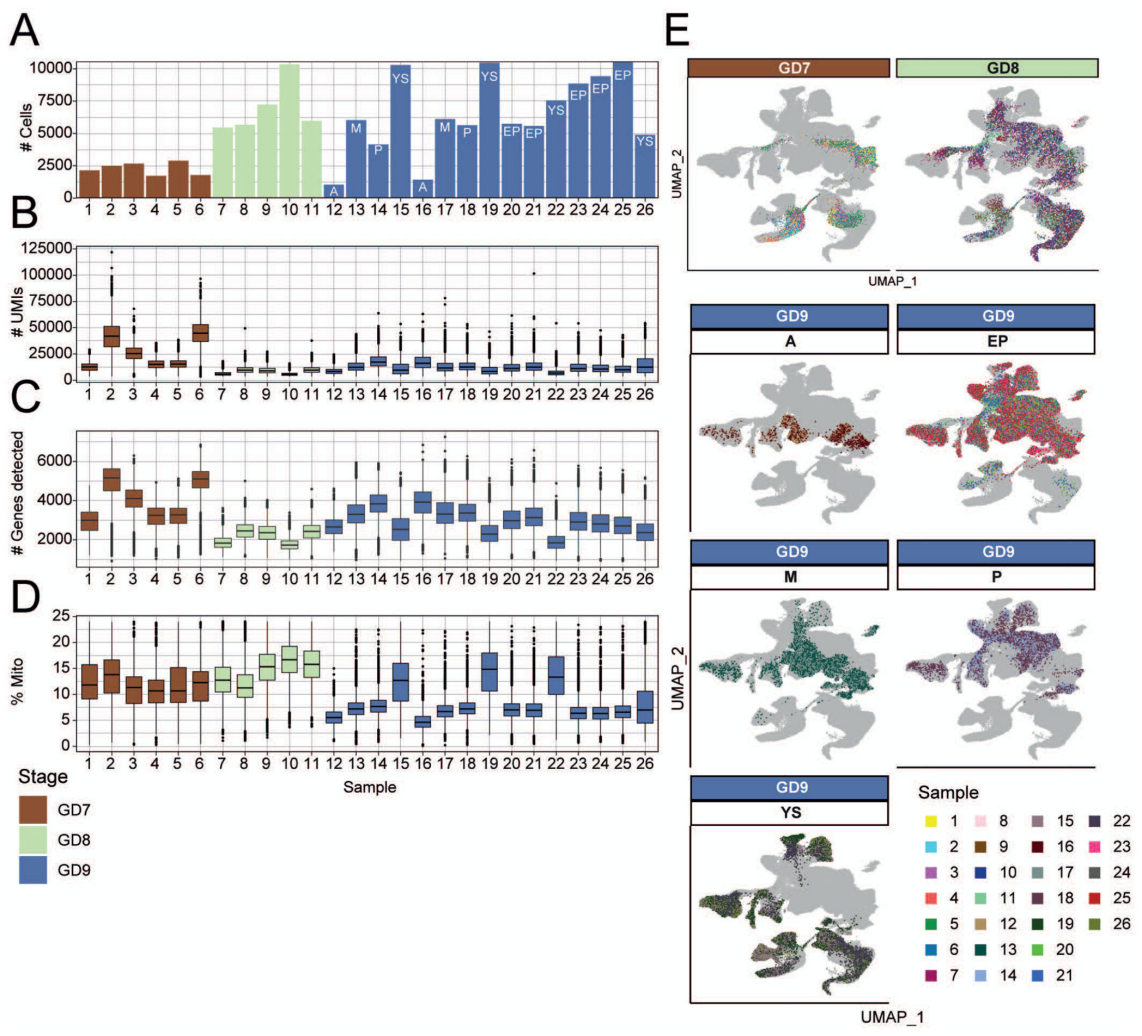


**Figure:**
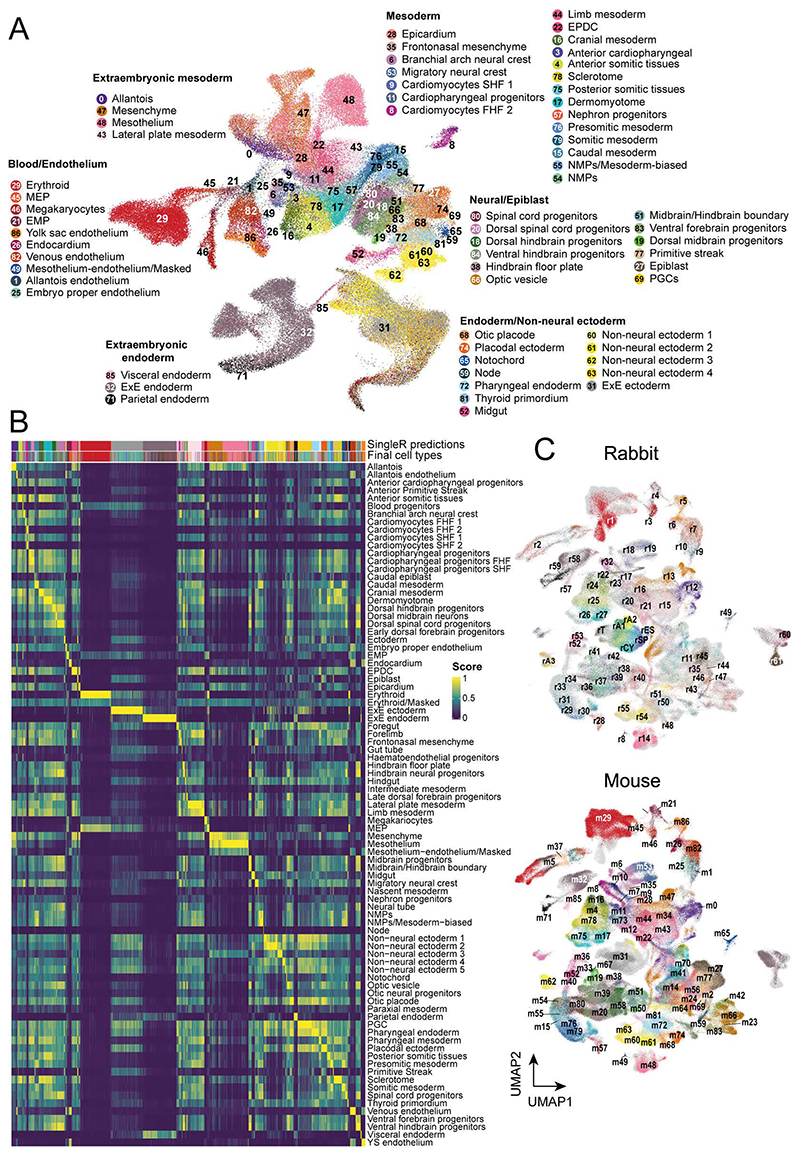


**Figure:**
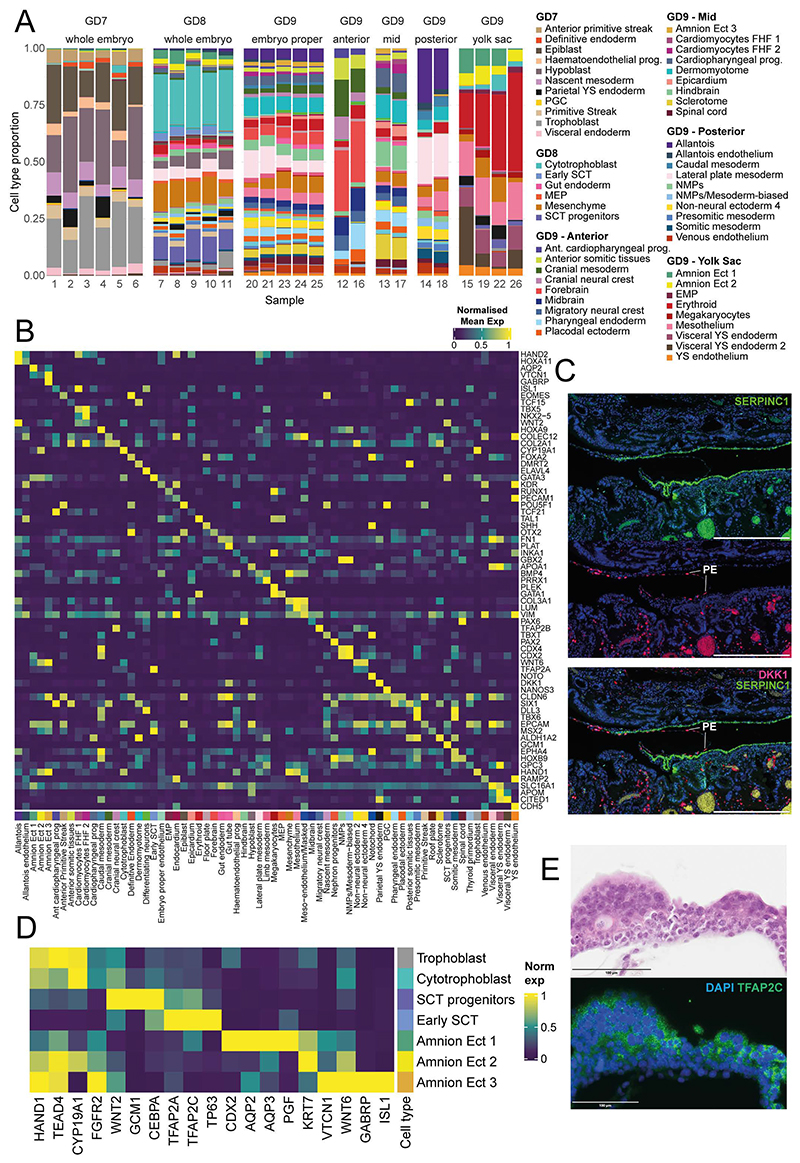


**Figure:**
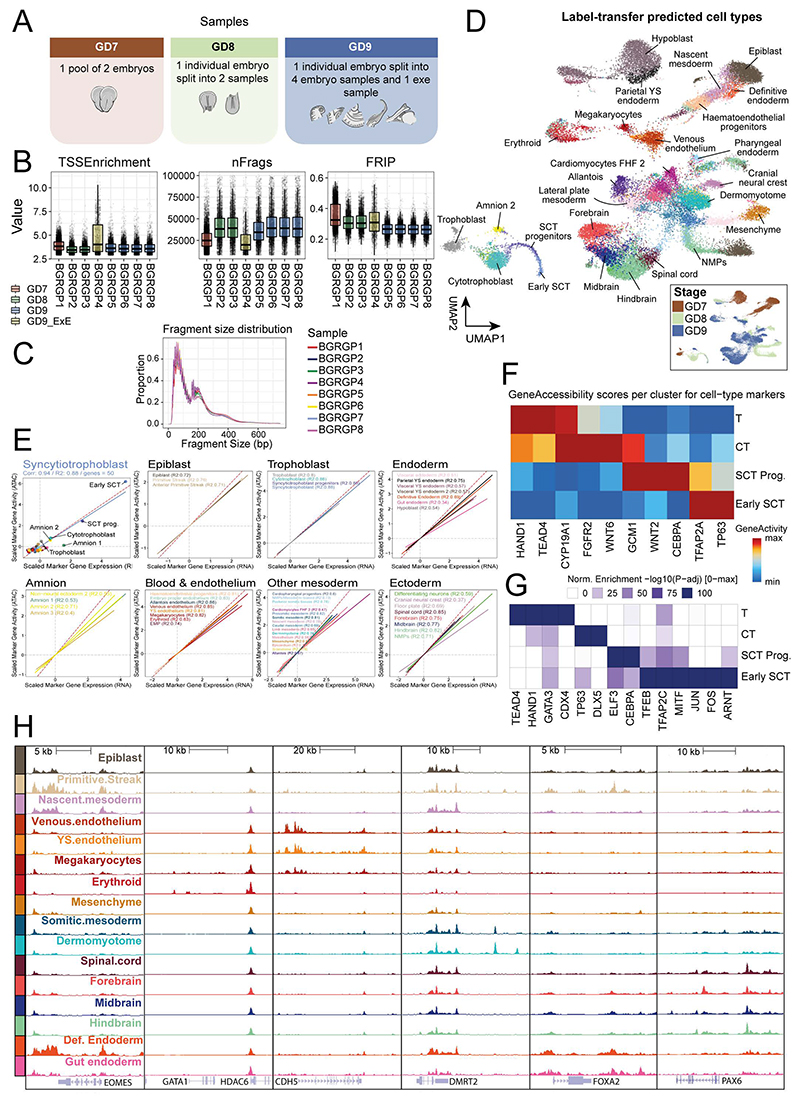


**Figure:**
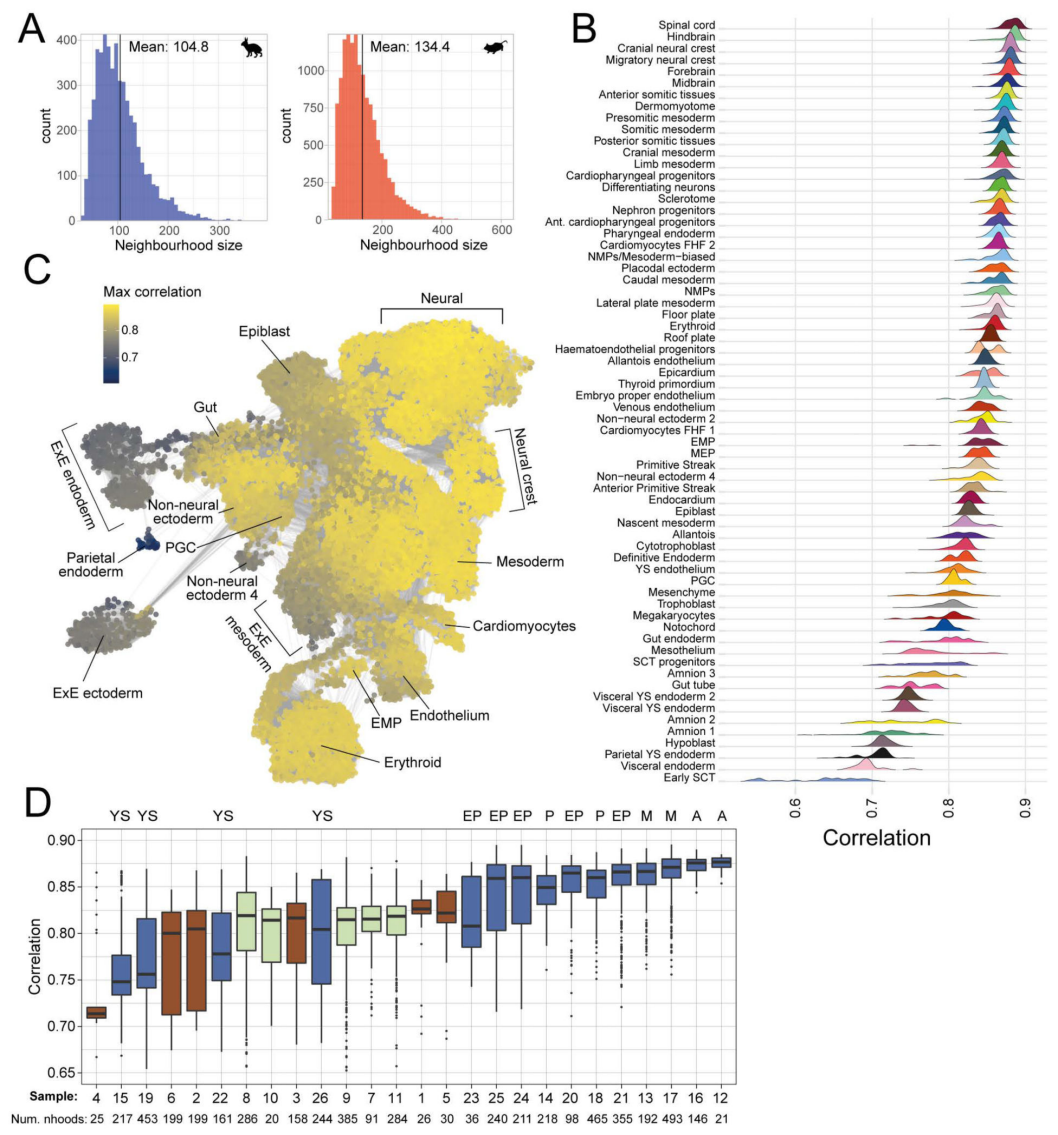


**Figure:**
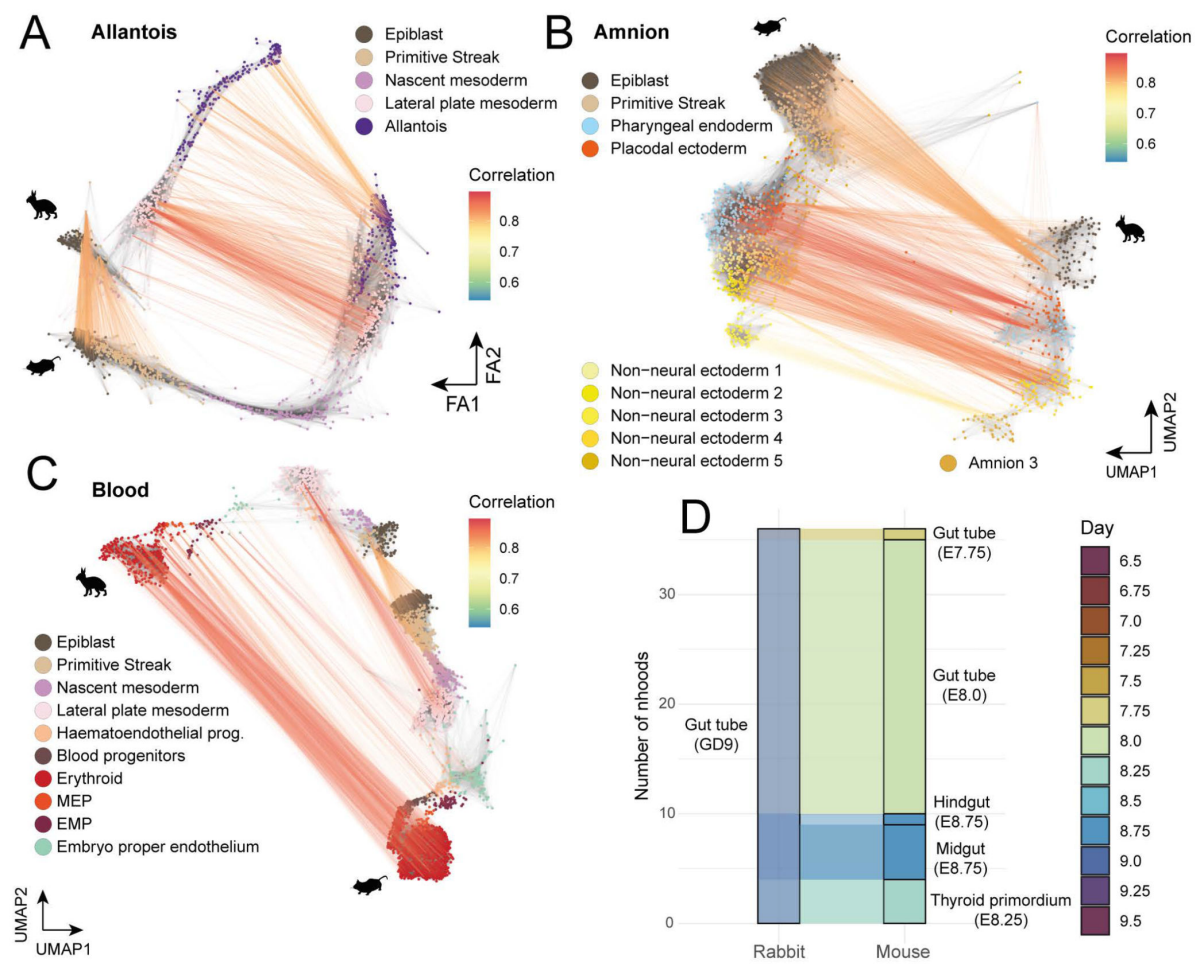


**Figure:**
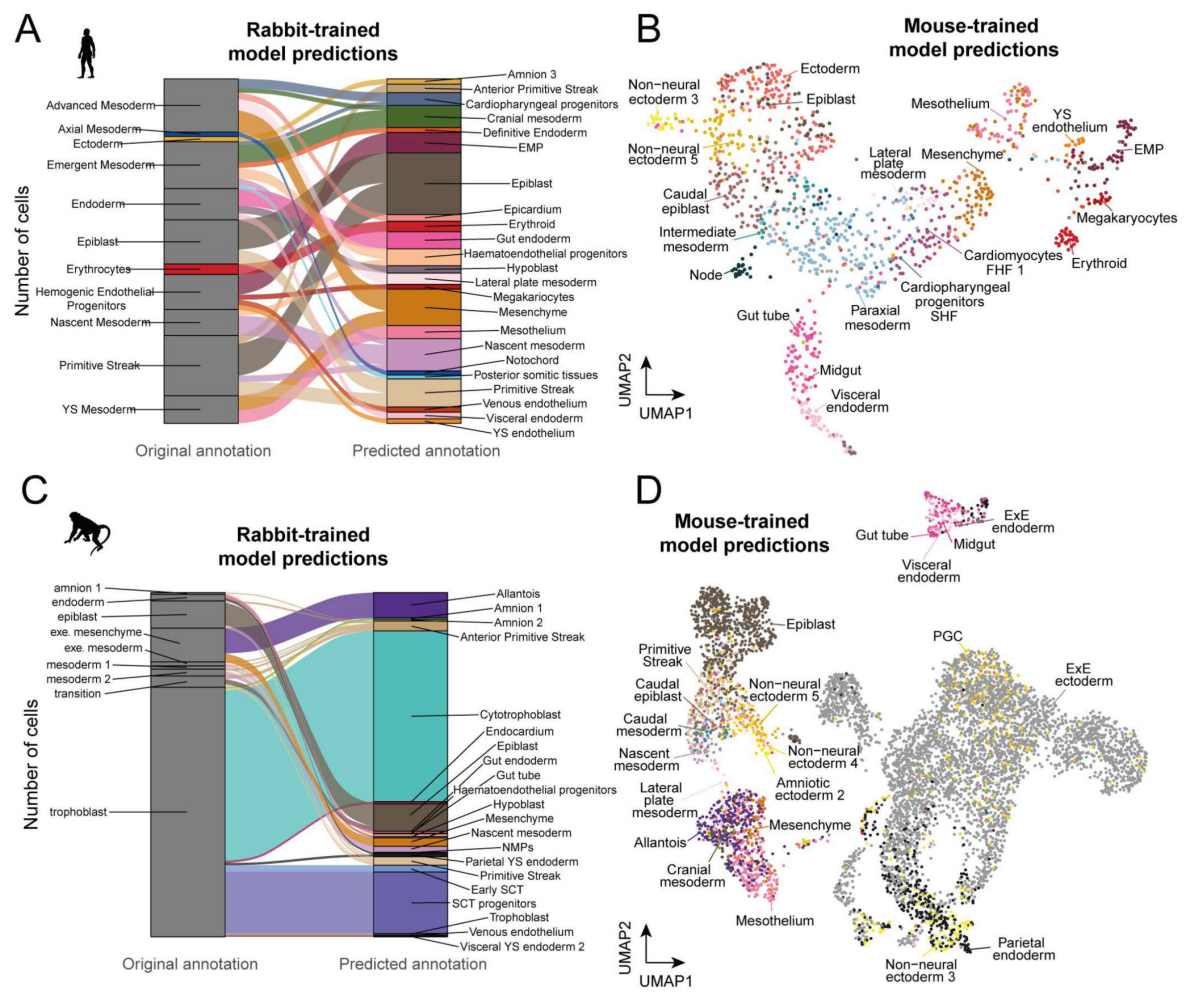


**Figure:**
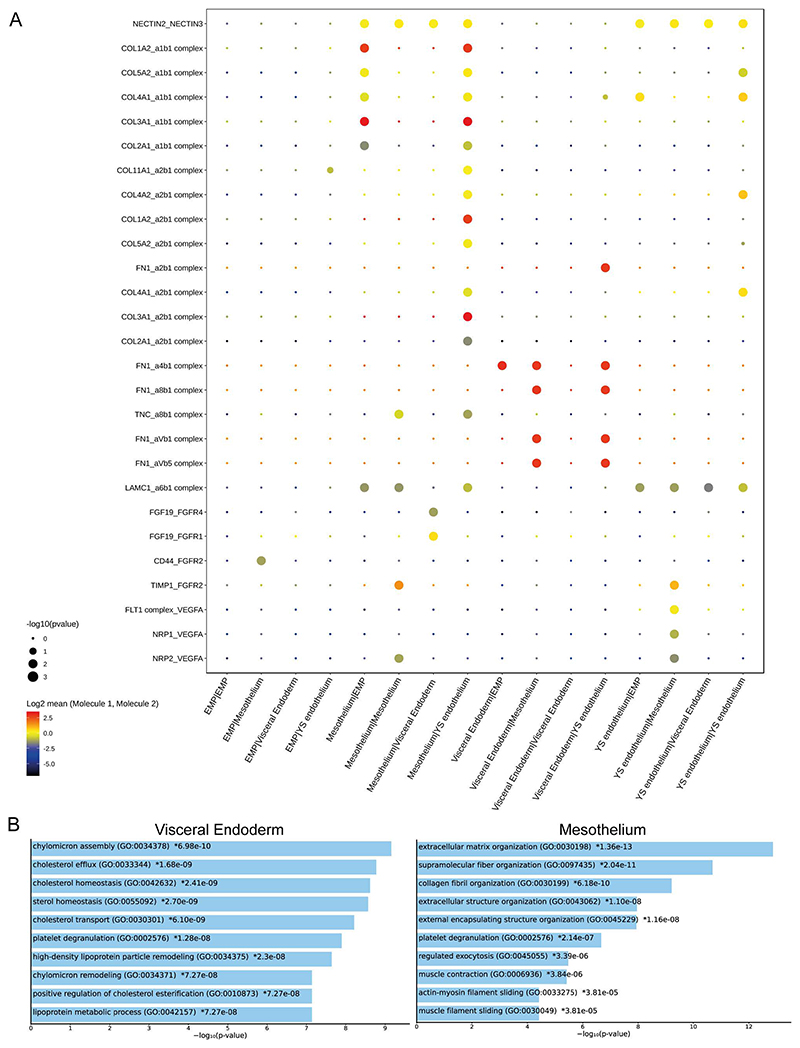


## Data Availability

Raw sequencing data are available through ArrayExpress with the following accessions: scRNA-seq: E-MTAB-11836; scATAC-seq: E-MTAB-11804. Raw histology and RNAscope imaging files are available through the EBI BioImage archive under accession S-BIAD604. All other links to the data are available at https://marionilab.github.io/RabbitGastrulation2022/. This includes links to processed single cell transcriptomics and ATAC-seq data in a variety of formats for loading into both R and python analysis pipelines. The transcriptomics and imaging data is also available to explore interactively via a web app accessible through the same link. Low-resolution thumbnail images for all generated imaging datasets are additionally provided. Source data have been provided in Source Data. All other data supporting the findings of this study are available from the corresponding author on reasonable request.

## Code Availability

Codes are available at https://github.com/MarioniLab/RabbitGastrulation2022 and https://marionilab.github.io/RabbitGastrulation2022/.
